# A genetic tool to express long fungal biosynthetic genes

**DOI:** 10.1186/s40694-023-00152-3

**Published:** 2023-02-01

**Authors:** Leo Kirchgaessner, Jacob M. Wurlitzer, Paula S. Seibold, Malik Rakhmanov, Markus Gressler

**Affiliations:** 1 Institute of Pharmacy, Department Pharmaceutical Microbiology, Friedrich Schiller University Jena, Winzerlaer Strasse 2, 07745 Jena, Germany; 2 Department Pharmaceutical Microbiology, Leibniz Institute for Natural Product Research and Infection Biology - Hans Knöll Institute, Winzerlaer Strasse 2, 07745 Jena, Germany; 3 Faculty Medical Technology and Biotechnology, Ernst Abbe University of Applied Sciences Jena, Carl-Zeiss-Promenade 2, 07745 Jena, Germany

**Keywords:** Early diverging fungi, Nonribosomal peptide synthetase, Polyketide synthase, Laetiporic acid, Calpinactam

## Abstract

**Background:**

Secondary metabolites (SMs) from mushroom-forming fungi (*Basidiomycota*) and early diverging fungi (EDF) such as *Mucoromycota* are scarcely investigated. In many cases, production of SMs is induced by unknown stress factors or is accompanied by seasonable developmental changes on fungal morphology. Moreover, many of these fungi are considered as non-culturable under laboratory conditions which impedes investigation into SM. In the post-genomic era, numerous novel SM genes have been identified especially from EDF. As most of them encode multi-module enzymes, these genes are usually long which limits cloning and heterologous expression in traditional hosts.

**Results:**

An expression system in *Aspergillus niger* is presented that is suitable for the production of SMs from both Basidiomycota and EDF. The *akuB* gene was deleted in the expression host *A. niger* ATNT∆*pyrG*, resulting in a deficient nonhomologous end-joining repair mechanism which in turn facilitates the targeted gene deletion via homologous recombination. The ∆*akuB* mutant tLK01 served as a platform to integrate overlapping DNA fragments of long SM genes into the *fwnA* locus required for the black pigmentation of conidia. This enables an easy discrimination of correct transformants by screening the transformation plates for fawn-colored colonies. Expression of the gene of interest (GOI) is induced dose-dependently by addition of doxycycline and is enhanced by the dual TetON/terrein synthase promoter system (ATNT) from *Aspergillus terreus*. We show that the 8 kb polyketide synthase gene *lpaA* from the basidiomycete *Laetiporus sulphureus* is correctly assembled from five overlapping DNA fragments and laetiporic acids are produced. In a second approach, we expressed the yet uncharacterized > 20 kb nonribosomal peptide synthetase gene *calA* from the EDF *Mortierella alpina*. Gene expression and subsequent LC–MS/MS analysis of mycelial extracts revealed the production of the antimycobacterial compound calpinactam. This is the first report on the heterologous production of a full-length SM multidomain enzyme from EDF.

**Conclusions:**

The system allows the assembly, targeted integration and expression of genes of > 20 kb size in *A. niger* in one single step. The system is suitable for evolutionary distantly related SM genes from both Basidiomycota and EDF. This uncovers new SM resources including genetically intractable or non-culturable fungi.

**Supplementary Information:**

The online version contains supplementary material available at 10.1186/s40694-023-00152-3.

## Introduction

Ascomycetes such as *Aspergilli* have become a powerful platform to heterologously biosynthesize secondary metabolites (SM) from various fungi [1]. Several classes of SM enzymes including polyketide synthases (PKSs), non-ribosomal peptide synthetases (NRPSs), PKS-NRPS hybrids, and terpene cyclases (TC) were successfully produced [2, 3]. However, fungal natural product research mainly focused on SM genes of manageable and cloneable size [4–7]. In contrast, longer genes (> 12 kb) have been preferably studied by either knock-out or promoter replacement strategies (or combination of both) [8], but have hardly been investigated by production in heterologous hosts. Both strategies require cultivability and transformability of the investigated fungi. These requirement are frequently met by Ascomycota, but are scarcely applicable to EDF such as *Mucoromycota* or higher fungi, i.e. *Basidiomycota* [9]. Hence, current knowledge on fungal secondary metabolite genes and their natural products is mostly based on work on ascomycetes and is thus biased. This is also founded on the elevated number of SM biosynthetic genes that have been identified in ascomycetes (10–30 NRPS and NRPS-like, 10–30 PKS, and > 4 TC genes per genome) *versus* basidiomycetes (1–6 NRPS and NRPS-like, 1–6 PKS, and 6–34 TC genes per genome) or EDF (0–15 NRPS and NRPS-likes, 0–15 PKS, 0–10 TC genes per genome) [9–12].

Several tools for the production of SM genes have been developed in ascomycetes. The heterologous expression of genes of interest (GOI) in *Aspergilli* require either a strong and constitutive promoter, such as the glycerinaldehyde-3-phosphate dehydrogenase promoter (P*gpdA*) [13] and the α-amylase promoter (P*amyB*) [14], or an inducible promoter system, such as the alcohol-inducible P*alcA*/*alcR* system [15], the tetracycline-dependent TetON system [16], or the ATNT system, i.e. a combination of the TetON system with the regulatory terrein biosynthetic promoter P*terA* [17, 18]. To date, both the P*alcA*/*alcR* system in *Aspergillus nidulans* and the ATNT system in *Aspergillus niger* are frequently used fungal expression platforms for genes encoding SM biosynthetic enzymes including PKSs [19, 20], NRPSs [21], and NRPS-like enzymes [22, 23]. However, alternative fungal systems have been developed, among them (i) the HEx platform using an engineered *Saccharomyces cerevisiae* strain producing additional SM auxiliary enzymes [24], (ii) the multiauxotrophic *Aspergillus oryzae* strains M2-3 and NSAR1 [25, 26], and various systems developed for *A. nidulans* including (iii) polyauxotrophic strains sensitive to various antibiotics [27], (iv) strains with strongly reduced intrinsic metabolic background [28, 29], (v) the nitrate-inducible *aflR/S* CoIN system [30], and (vi) an AfoA-inducible platform [31]. Traditionally, single SM biosynthetic genes were expressed in phylogenetically distantly related hosts such as *Escherichia coli* and *S. cerevisiae* [4, 32]. However, the latter two platforms require adaptations and adjustments such as (i) the co-production of SM specific phosphopantetheinyl transferases to produce holoenzymes [33], or (ii) the expression of additional tRNAs to ensure active enzymes of sufficient yield [34] and, moreover, may fail due to limitations of intron splicing [24]. In any case and prior to transformation of the heterologous host, the GOI must be amplified from DNA and cloned in plasmids that are usually assembled and propagated in *E. coli* or *S. cerevisiae* [35, 36].

Fungal genes encoding highly reducing PKSs (8–9 kb), PKS-NRPS hybrids (12 kb) and multimodule NRPSs (10–26 kb) are of extraordinary size and can be even longer if interspaced by introns. Hence, a full-length amplification by PCR using genomic DNA as template is not recommendable. An amplification from cDNA is the gold standard, but is impaired as: (i) reverse transcriptases possess low processivities at long templates [37], and (ii) most fungal SM genes remain silent under standard laboratory conditions—a fact, which reduces the availability of a full-length RNA template [38]. Hence, an amplification and cloning in small fragments is a conceivable way to manage long GOI. Since 1990 artificial chromosomes (FACs, YACs and BACs) has been frequently used [39, 40], and are suitable to express entire gene clusters of 50–150 kb size. [40] However, identification and metabolic screening of correct clones is laborious and time-consuming. Moreover, FACs and BACs rely on an intrinsic activation of the transgenic promoters in the expression host which is hardly achieved for genes from distantly related species.

Here, an alternative strategy to clone and express long GOI is presented, which is based on the well-established ATNT expression system in *A. niger* and does not require cloning of the full-length gene. Instead, the GOI is amplified in up to five fragments and is assembled with high fidelity by homologous recombination within the host in one single step. On-site integration into the *fwnA* locus responsible for conidial pigmentation allowed a simple visual screening for the correct transformants. The system’s versatility was proven by the integration of two GOI from phylogenetically divergent fungi: First, the *lpaA* gene (8.2 kb) from the basidiomycete *Laetiporus sulphureus*, known to encode a PKS responsible for the chromophoric laetiporic acids [41], was precisely integrated in five DNA fragments. Furthermore, an unknown NRPS gene (*calA,* 20.0 kb) from the EDF *Mortierella alpina* was successfully expressed and assigned as a synthetase for calpinactam, an anti-mycobacterial peptide. Hence, the system sets the basis to study long biosynthetic genes from various biological sources including EDF.

## Results

### Deletion of the *akuB* gene in ATNT favors homologous recombination

**Fig. 1:**
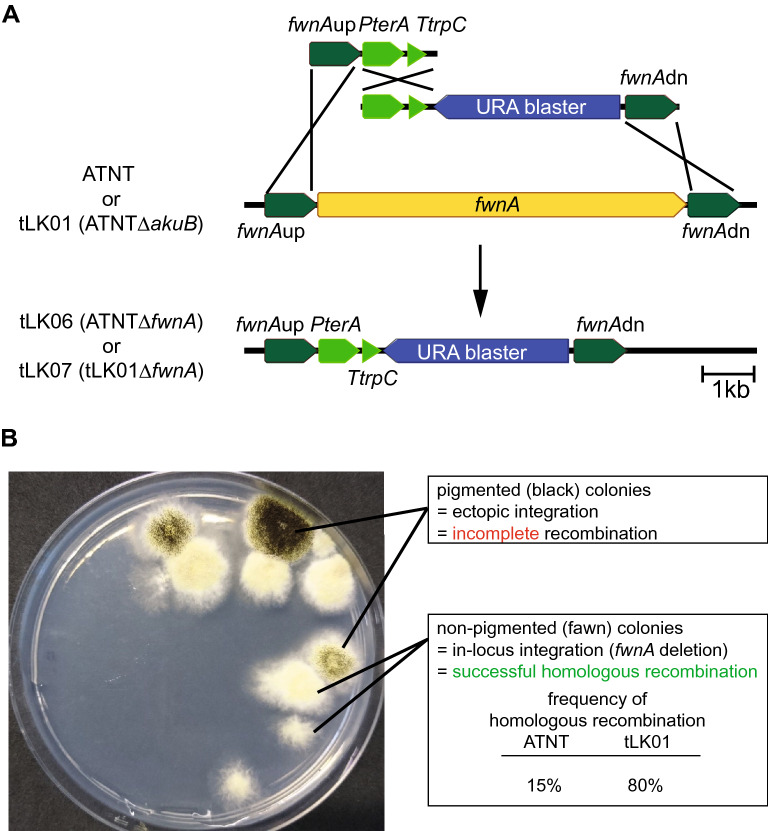
Principle of the recombination and ∆*fwnA* selection system. **A** Construction of the *fwnA* deletion mutants in the parental strains *A. niger* ATNT and tLK01 (ATNT∆*akuB*). Homologous recombination of the two PCR amplicons into the *fwnA* locus in the parental strains led to the deletion of the *fwnA* gene resulting in *A. niger* strains tLK06 (ATNT∆*fwnA*) and tLK07 (tLK01∆*fwnA*). **B** Representative agar plate of a transformation of the *A. niger* tLK01 parental strain with the split-marker *fwnA* deletion constructs. Non-pigmented, fawn colonies are resulting from the *fwnA* deletion suggesting a successful recombination event whilst pigmented, black colonies indicate false-positive transformants. The frequency of homologous recombination is significantly increased in the *A. niger* ∆*akuB* deletion strain tLK01

*Aspergilli* randomly integrate transgenes into their genomes and multiple integration events are frequently observed [42]. It has been demonstrated, that *Aspergillus* or *Neurospora* deletion mutants defective in the non-homologous end-joining (NHEJ) repair mechanism such as ∆*akuA*^KU70^,∆*akuB*^KU80^, or ∆*ligA,* facilitate the targeted gene deletion by homologous recombination [43–45]. Moreover, these mutants can integrate transgenes in two overlapping fragments [29]. This “split-marker” recombination is broadly used for efficient targeted gene deletion in fungi [46]. To investigate the applicability of a deletion of the NHEJ system for a targeted multi-fragment gene integration, the *akuB* gene from the *A. niger* expression strain ATNT∆*pyrG* was replaced by the hygromycin B resistance cassette and the *akuB* deletion in the obtained mutant *A. niger* ATNT∆*akuB* (tLK01) was confirmed by Southern Blot (Additional file 5: Fig. S1). To test the frequency of transgene integration, the *fwnA*^*albA*/*pksP*^ gene encoding the polyketide synthase responsible for conidial pigmentation [47] was deleted in both *A. niger* ATNT and mutant tLK01 (Fig. 1A). To this end, the overexpression plasmid pPS01 [41] containing the inducible promoter P*terA*, the terminator T*trpC,* and an URA blaster was expanded by the upstream and downstream flanks of *fwnA* (5′ *fwnA*up and 3′ *fwnA*dn) resulting in expression plasmid pLK04 (Additional file 6: Fig. S2)*.* When both ATNT and tLK01 were transformed with the overlapping fragments of the construct, the frequency of targeted recombination was significantly higher for tLK01, i.e. the relative number of non-pigmented transformants was increased from 15 to 80% when compared to the parental strain (Fig. 1B, Additional file 2: Table S1, Additional file 7: Fig. S3). A successful genomic integration was additionally confirmed by PCR (Additional file 8: Fig. S4). One of these *A. niger* tLK01∆*fwnA* transformants (tLK07) served as null mutant (empty vector control) in subsequent metabolite analyses.

### *Aspergillus niger* tLK01 is competent to integrate five-fragment expression constructs

**Fig. 2:**
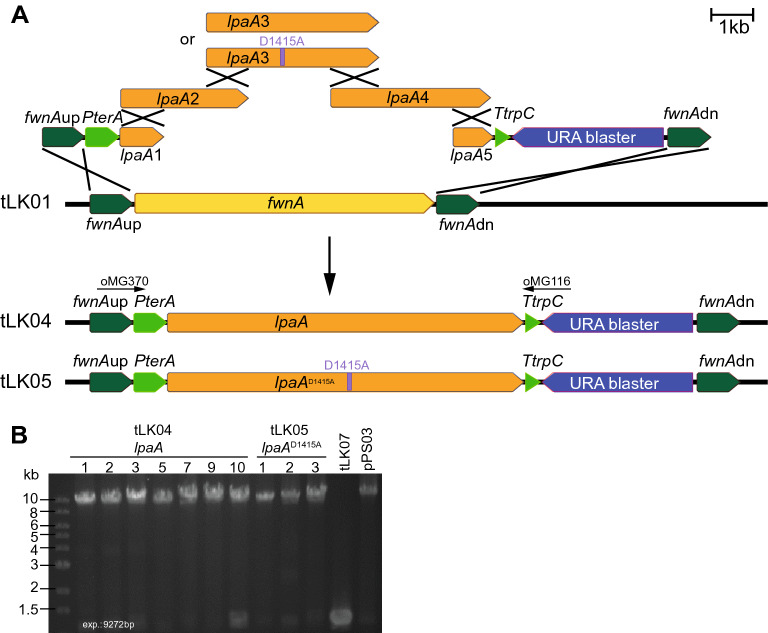
Determination of the full-length integration of the P*terA*:*lpaA:*T*trpC* construct into the *fwnA* genomic locus of the *A. niger* recipient strain tLK01. **A** Schematic representation of the integration event of the five *lpaA* fragments into the genomic *fwnA* locus of *A. niger* tLK01. To mutate the SAM binding site in the C-methyltransferase domain of LpaA, the triplet GAC (pos. 4245–4247) encoding Asp^1415^ (probably binding the ribose moiety of SAM) was site-mutated into the triplet GCC encoding Ala^1415^. **B** An agarose gel of a PCR targeting the P*terA* promoter and the T*trpC* terminator has been performed (oligonucleotides oMG370/oMG116). The expected amplicon size is indicated. Full length integration was evident for seven tLK04 (producing native LpaA) and three tLK05 transformants (producing LpaA^D1415A^). The genomic DNA of tLK07 (transformed with an empty vector) and the *lpaA*-encoding plasmid pPS03 [41] served as negative and positive controls, respectively

**Fig. 3:**
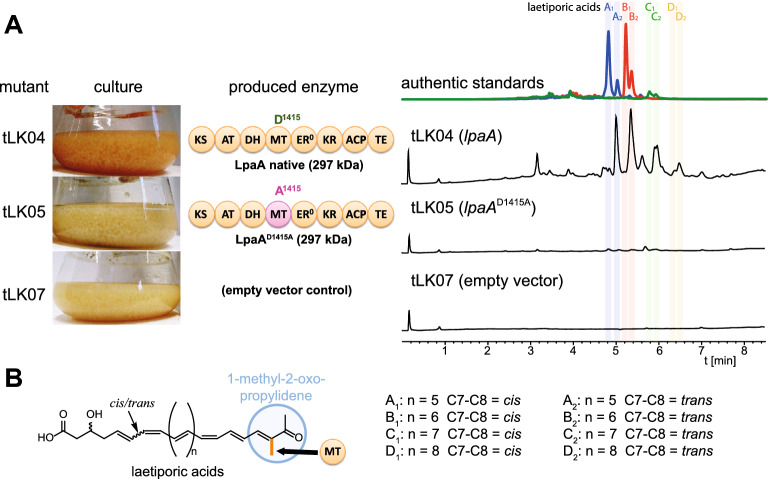
Laetiporic acid production in the *A. niger* ∆*fwnA* mutants tLK04, tLK05 and tLK07.** A** Cultures photographs, representative domain structure of LpaA mutant proteins and HPLC profiles of metabolite extracts. Photographs depict 3-days cultures at 30 °C, 150 rpm. HPLC chromatograms of extracts of mycelia from tLK04, tLK05 and tLK07 were monitored at a wavelength of λ = 450 nm. Note, that tLK04 expressing the native *lpaA* gene produces laetiporic acids A_1_–D_2_, whereas no metabolites are detectable in tLK05 (expressing the mutant *lpaA*^D1415A^ gene) or in tLK07 (empty vector control). Authentic standards of laetiporic acids (LA-A_1/2_, LA-B_1/2_ and LA-C_1/2_) served as controls. An authentic standard for LA-D_1/2_ was not available. For MS and MS/MS spectra please refer to Additional file 10: Fig. S6. The pearls on a string represent the domain structure of the PKS LpaA and include: KS, β-ketoacyl synthase; AT, acyl transferase; DH, dehydratase; MT, C-methyltransferase; ER^0^, (inactive) enoyl reductase; KR, keto reductase; ACP, acyl carrier protein; TE, thioesterase. **B** Chemical structure of laetiporic acids A_1_–D_2_. Note the common 1-methyl-2-oxo-propylidene group, which requires the action of the intrinsic C-MT domain

As a proof of concept to integrate long SM genes in the *fwnA* locus of *A. niger* tLK01 by application of multiple DNA fragments, we used the 8196 bp PKS gene *lpaA* from the “chicken of the woods” mushroom *L. sulphureus* [41]. Recently, this gene has been successfully expressed in *Aspergilli* and was assigned to the biosynthesis of polyenes of various chain lengths (C_26_–C_32_), i.e. laetiporic acids A_1_-D_1_ (LA A_1_–D_1_) and their 7-*trans*-isomers A_2_ -D_2_ (LA A_2_–D_2_) [41], which both are currently discussed to be natural alternative colorants in cosmetics and for textiles [48]. Due to their absorption maxima between 450 and 470 nm, the mycelium of producing cultures exhibit an orange hue allowing an easy read-out of positive transformants. We fused a 1 kb fragment of either end of *lpaA* and ligated the fusion fragment into pLK04. Five fragments with a 1 kb overlap were amplified by PCR (Fig. 2A), mixed equimolarly and used to transform *A. niger* tLK01 using uridine prototrophy as selection marker. Five non-pigmented transformants were randomly picked and checked by PCR for the full-length integration of the expression construct (Fig. 2B) and by Southern Blot for homologous integration into the *fwnA* locus (Additional file 9: Fig. S5). Indeed, four out of five fawn tLK04 transformants (4/5, 80%) successfully assembled and integrated the *lpaA* expression construct into the *fwnA* locus. Finally, the transformant tLK04 and the respective empty vector control strain tLK07 were fermented under non-inducing and inducing conditions (by addition of doxycycline). Metabolites were extracted and analyzed by UHPLC-MS (Fig. 3A) and by high resolution MS fragmentation (Additional file 10: Fig. S6). During cultivation the color of the fungal mycelium turned orange and the expected polyene products LA A_1_–D_2_ were primarily detectable from mycelium of the doxycycline-induced cultures of tLK04 suggesting that the five DNA fragments have been accurately assembled. Overall, seven out of seven PCR-positive transformants (Fig. 2B) produced the expected compounds (7/7, 100%). Minor signals of LAs with 100-fold less abundances were detectable from non-induced cultures, suggesting that the promoter is not entirely silent (not shown). In addition, no laetiporic acids were obtained from the empty vector control tLK07 under inducing conditions (Fig. 3A).

### The expression system is suitable for point mutation analysis

Next, the suitability of system to study point mutations of long GOI was investigated. Structurally, linear polyenes including the antifungal laetiporic acids from *L. sulphureus* [41], piptoporic acid from curry punk fungus *Piptoporus australiensis* [49]*,* and the two antilarval polyenes from the undescribed stereaceous basidiomycete BY1 [50, 51], feature a 1-methyl-2-oxo-propylidene head as plausible pharmacophore (Fig. 3B). We speculated on the intrinsic activity of the C-methyl transferase (C-MT) domain in LpaA to catalyze this *S*-adenosyl-methionine (SAM) dependent C-transfer after two cycles of acyl condensation. Hence, we aimed at the inactivation of the C-MT domain. In a previous study, an aspartate (D^2019^) in the active site of citrinin polyketide synthase PksCT was shown to be essential for the methylation of the polyketide citrinin by coordination of 2′-OH and 3′-OH of the ribose moiety of the cofactor SAM [52]. Hence, to inactive the C-MT activity of LpaA, the GAC triplett encoding the homolog aspartate (D^1415^) was mutated into GCC (A^1415^). To this end, five DNA fragments were used to transform *A. niger* tLK01, of which fragment 3 contained the mutated sequence encoding the potential dysfunctional MT^D1415A^ (Fig. 2A). Full-length integration of *lpaA*^D1415A^ was confirmed by PCR (Fig. 2B) and the point mutation was additionally confirmed by Sanger sequencing. Surprisingly, whilst cultures of the *lpaA*-expressing strain tLK04 turned orange after induction, the induced transformants expressing the *lpaA*^D1415A^ gene (tLK05) neither changed mycelial color nor produced any additional compound when compared to the empty vector control (tLK07) (Fig. 3A). This observation is not based on an altered transcription because *lpaA* in tLK04 and *lpaA*^D1415A^ in tLK05 showed identical expression levels (Additional file 11: Fig. S7). Hence, the C-MT seemed to be essential for polyketide production. This finding in turn, implies that the 1-methyl-2-oxo-propylidene head in laetiporic acids is required for a successful chain elongation. In sum, the strain tLK01 is convenient to assemble DNA constructs from up to five fragments and directly express the GOI in one step. Moreover, the system is suitable to induce point mutations in long GOIs to study modified enzymes.

### The NRPS gene *calA* encodes the calpinactam synthetase in *Mortierella alpina*

**Fig. 4:**
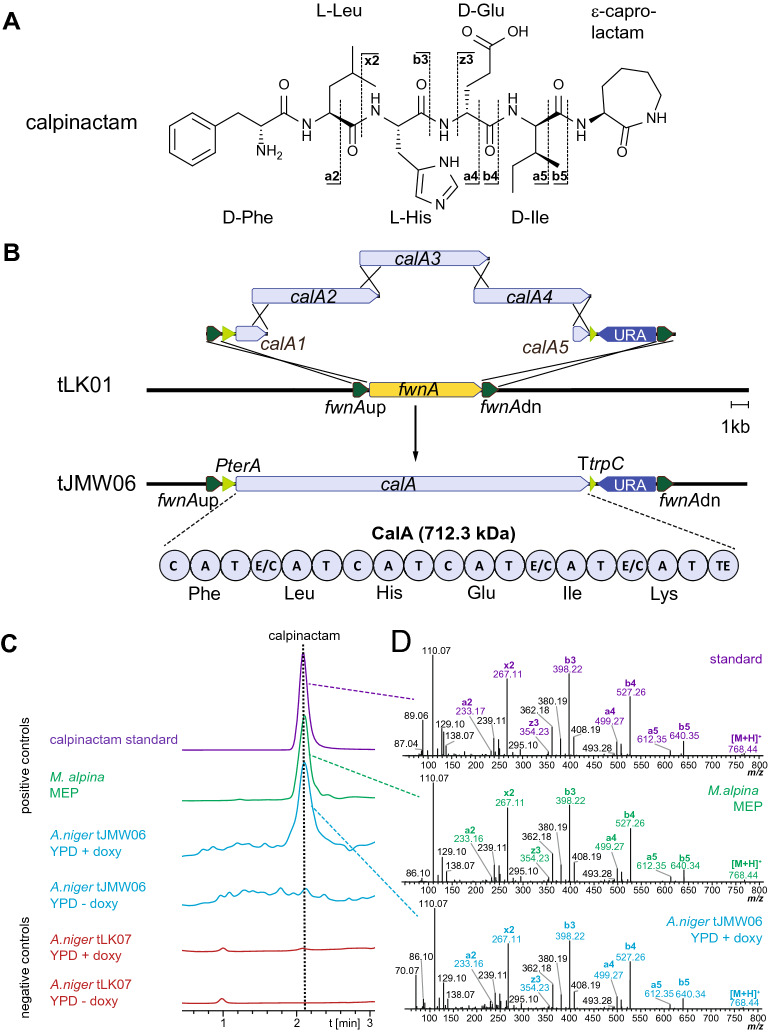
Calpinactam production in *M. alpina* and transgenic *A. niger* ∆*fwnA* mutants tJMW06 and tLK07.** A** Chemical structure and calculated MS/MS fragments of calpinactam.** B** Schematic representation of the integration event of the five *calA* fragments into the genomic *fwnA* locus of *A. niger* tLK01. The pearls on a string represent the domain structure of the 6-module NRPS CalA and include: C, condensation domain; A, adenylation domain; T, thiolation domain; E/C dual epimerization/condensation domain; TE, thioesterase. Note that the 1st (starter) C domain is truncated and inactive. **C** Extracted ion chromatograms of the synthesized calpinactam standard and metabolic extracts from various fungal mycelia. *M. alpina* ATCC32222 was cultivated on MEP, whilst transgenic *A. niger* tJMW06 (*calA* expressing) and tLK07 (empty vector control) was cultivated in *Aspergillus* Minimal Medium (AMM) under inducing (+ doxycycline) or non-inducing (− doxycycline) conditions. MS data was monitored in positive ionization mode at *m/z* 768.6844 [*M* + H]^+^. **D** MS/MS spectra highlighting the specific daughter ion fragments of calpinactam of the authentic standard and the metabolic extracts from *M. alpina* ATCC32222 and transgenic *A. niger* tJMW06

In contrast to Dikarya, early diverging fungi (EDF) are a comparably new resource of natural products. Although the genomes of *Mortierella *sp. (Phylum *Mucoromycota*) and *Basidiobolus *sp. (Phylum *Zoopagomycota*) encode numerous of long NRPS genes (up to 26 kb), only a few have been assigned to specific products [10]. Calpinactam, produced by *M. alpina* FKI-4905*,* is a hexapeptide with an unusual C-terminal ε-caprolactam moiety (Fig. 4A) [53]. Calpinactam—but not its chemically closely related derivatives [54, 55]—is a proven anti-mycobacterial agent with MICs of 0.78 µg mL^−1^ and 12.5 µg mL^−1^ against *Mycobacterium smegmatis* or *Mycobacterium tuberculosis,* respectively [53]. Although of high pharmaceutical interest, the corresponding gene for calpinactam biosynthesis has never been identified. We examined the metabolome and genome of the *M. alpina* sequencing reference strain ATCC32222 [56]. The strain produces and secretes calpinactam into the supernatant as confirmed by UHPLC-MS and comparative MS–MS fragmentation patterns versus a commercially available authentic standard (Fig. 4C and D). Three hexamodular candidate NRPS genes (*nps5*, *nps6* and *nps7*) were identified in the *M. alpina* genome [57]. Since *Mortierella* NRPS genes are likely of bacterial origin [58], we predicted the substrate specificity codes of the candidate enzymes versus the bacterial gramicidin S synthetase GrsA from *Aneurinibacillus migulanus* as reference (Table 1). While at least three of the adenylation (A) domains encoded by *nps6* or *nps7* are expected to use identical amino acids, the six A domains encoded by *nps5* (*calA*) accept six dissimilar substrates as required for calpinactam (Fig. 4A, Table 1). Moreover, the arrangement of condensation (C) and dual epimerization/condensation (E/C) domains in CalA directly reflects the sequence of l- and d-amino acids in the final peptide chain of calpinactam according to the NRPS collinearity principle [59] (Table 2). Hence, the postulated domain structure and the predicted A domain substrate specificities of CalA perfectly matched an NRPS assembly line for calpinactam.

**Table 1:**
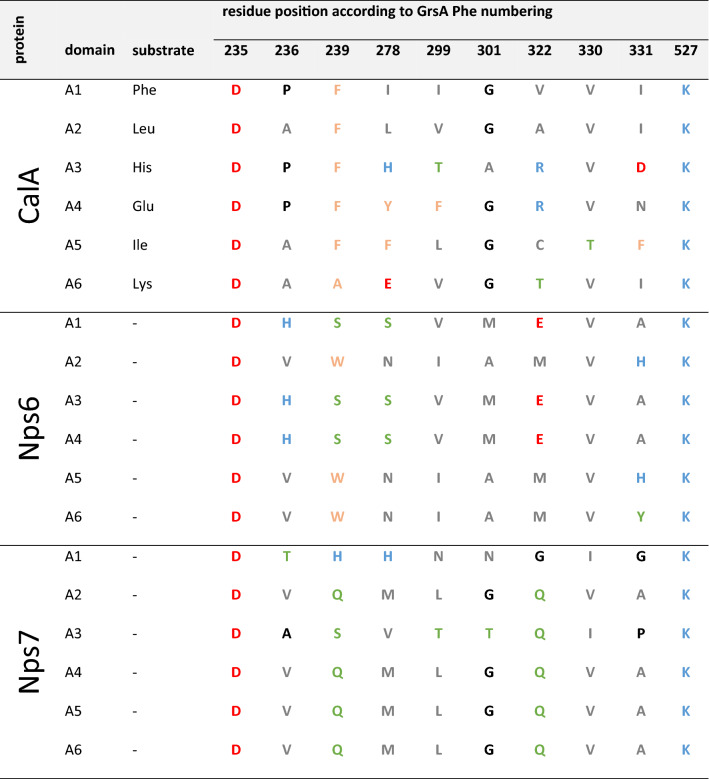
Substrate specificity code for adenylation domains of hexamodular NRPSs of *Mortierella alpina*

The residues are mapped based on positions according to *Aneurinibacillus migulanus* (former *Brevibacillus brevis*) GrsA-A numbering [101]. Amino acid residues in the NRPS code are highlighted according to their physicochemical properties: acidic (red), small/hydrophobic (grey), aromatic/hydrophobic (ocher), hydrophilic (green), and basic (blue)

**Table 2:**
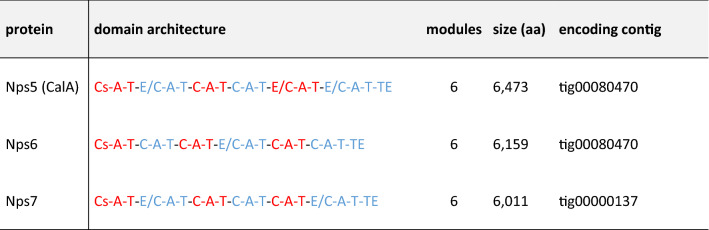
Domain architecture of predicted hexamodular NRPSs in *M.alpina*

For clarity, individual modules are highlighted in alternating red and blue color. A, adenylation domain; C, condensation domain; Cs (inactive) starter condensation domain; E/C, dual epimerization/condensation domain; TE, thioesterase domain

### Full-length *calA* gene expression in* A. niger* enables a heterologous calpinactam production

Given its length of 20,015 bp, we decided to heterologously express *calA* from *M. alpina* in *A. niger* tLK01 in a similar fashion as *lpaA* using collectively five *calA* DNA fragments of up to 7000 bp (Fig. 4B). 30 transformants were obtained of which three randomly picked transformants (tJMW06.3, tJMW06.13, tJMW06.26) showed a full-length integration of *calA* into the genome of *A. niger* as confirmed by multiple PCRs (Additional file 12: Fig. S8) and by Southern Blot (Additional file 13: Fig. S9). Profiling the metabolome of the tJMW06 transformants, an additional metabolite was detected under inducing conditions, whose ion mass (*m/z* 768.4411 [*M* + H]^+^) and retention time (2.1 min) was identical to that of the commercial calpinactam standard (MW 768.4330 Da) (Fig. 4C). The identical MS/MS-fragmentation pattern confirmed the heterologous production of calpinactam (Fig. 4D). In contrast, calpinactam was not detectable in the empty vector control strain tLK07 (Fig. 4C). Hence, we established *calA* as the calpinactam biosynthetic gene.

**Fig. 5:**
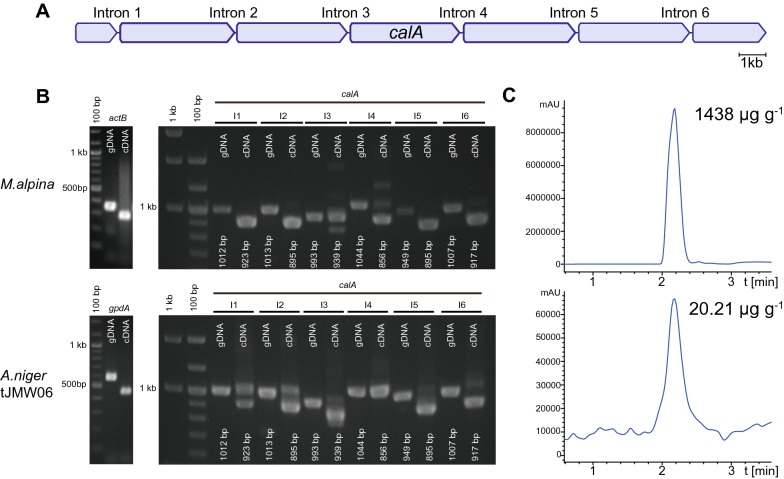
Splicing events in the *calA* gene in *M. alpina* and transgenic *A. niger* tJMW06.** A** Structure of the full-length 20 kb *calA* gene. The gene is disrupted by six introns.** B** Splicing pattern of natively expressed *calA* in *M. alpina* and heterologously expressed *calA* in *A. niger* tJMW06. Both strains were cultivated under inducing conditions (*M. alpina* in MEP medium, *A. niger* tJMW06 in AMM with doxycycline). Genomic (gDNA) and cDNA served as templates for diagnostic PCRs spanning the intron in a housekeeping gene (*actB* or *gpdA*, respectively) or individually spanning the six introns of *calA*. Expected fragment sizes are indicated. **C** MS profile at *m/z* 768.44 [*M* + H]^+^ of the induced cultures from *M. alpina* (upper lane) and *A. niger* tJMW06 (lower lane). The indicated titer of calpinactam is normalized to the fungal dry weight (calculated from three replicates)

The production of calpinactam in *A. niger* tJMW06 was highest (20 µg g^−1^ fungal dry weight), when the transgenic fungus was cultivated at 25 °C (Additional file 14: Fig. S10), i.e. the temperature optimum for *M. alpina* enzymes [60]. However, titers of calpinactam remained low compared to the original producer strain *M. alpina* (1438 µg g^−1^). The > 20 kb *calA* gene includes six potential introns of 90 to 112 bp length (Fig. 5A). Hence, we checked for the correct splicing pattern of the *calA* transcript by comparative PCR on the *M. alpina* and *A. niger* tJMW06 cDNA (Fig. 5B). The complete set of introns were spliced in its original producer strain *M. alpina* as predicted*,* although introns 3 and 4 showed only partial splicing*.* Similarly*,* the splicing of introns 1, 2, 3, 5 and 6 appeared at the correct sites in the transgenic *A. niger* tJMW06. However, only marginal pre-mRNA maturation was detectable for intron 4, as genomic DNA and cDNA show the same PCR fragment sizes (Fig. 5B). The unspliced intron 4 would result in a premature termination at a UAG stop codon at position 11,148. Hence, this incomplete splicing event results in a loss-of-function version of CalA and may explain the low calpinactam titers in *A. niger* tJMW06 (Fig. 5C). To bypass this limitation, we removed the intron 4 by amplification and PCR-fusion of the adjacent exons of fragment 3 and transformed the recipient strain *A. niger* tLK01 with the altered version of *calA* fragments. However, this manipulation did not change the titers (data not shown), suggesting that additional adjustments such as codon-optimization and balanced supply of precursors might be required for increased calpinactam titers. In summary, in this pilot study we established for the first time a successful five-fragment assembly and heterologous expression of a > 20 kb natural product biosynthetic gene from an early diverging fungus.

## Discussion

Over the past four decades, 60% of all new antibiotic lead structures were based on natural products [61]. Although new antibiotics are urgently needed especially to treat nosocomial infections, natural product research slowed down as mainly bacteria and ascomycetes were investigated concerning their metabolic potential and rediscovery of known compounds is frequently observed [62]. In contrast basidiomycetes and especially EDF are an attractive resource of bioactive agents but are rather genetically uninvestigated which is due to their challenging cultivability and the lack of genetic accessibility [9, 63]. Here, we present a molecular tool to express entire long SM genes to unravel the metabolic treasure chest of these fungi.

Multiple gene expression tools have been developed for ascomycetes (genera *Aspergillus*, *Fusarium*, *Penicillium*, *Pichia* and *Saccharomyces*) and partially for basidiomycetes (genera *Ustilago, Coprinus*). No system for EDF has been established yet. Although anectodical evidence for their transformability exists using the more complex molecular tools such as CRISPR/Cas9 or TALEN/exonuclease [64–66], no common strategy for a targeted gene deletion exists since the *ku70/80* homologs are generally not expressed in these fungi [64]. Hence, unrevealing the function of the SM biosynthetic genes from Basidiomycota and EDF still rely on heterologous expression. However, main issue is the size of the SM genes and its interruption with introns which impedes an expression in traditional bacterial hosts [9]. Several molecular tools to clone and express entire bacterial gene clusters of > 40 kb were developed in *Streptomyces* species [67]. However, the current fungal expression systems mainly vary in their type of promoter activation, but most of them require are a pre-cloning of the GOI into a specific vector, which limits their applicability to long SM biosynthetic genes. Effort was done with the yeast/*E.coli* shuttle vectors in yeast, allowing a multi-gene assembly and co-expression of smaller biosynthetic genes in yeast and *Aspergillus* [68–70]. Alternatively, reiterative recombination systems assemble multigene constructs in *S. cerevisiae*, but this endonuclease-induced recombination requires multiple rounds of transformation to recycle selectable markers and is hence a robust, but more laborious technique [71]. Moreover, the genes of *S. cerevisiae* rarely possess introns (0.04 introns per gene) in comparison to *Aspergillus* species (0.96) making yeast to a less suitable host for multiple spliced transgenes [72].

The herein described system combines current knowledge of fungal biotechnology including the higher recombination frequency by deletion of the *akuB* gene [73], the easy transformability of *Aspergilli* [27], the discrimination of positive transformants by altered pigmentation [29] and the possibility to express > 20 kb genes in *Aspergilli* [16, 18]. The method is advantageous since (i) genomic DNA can be used as template, (ii) cloning steps are set to a minimum, (iii) assembly and expression of long genes is accomplished in a single step, and (iv) gene expression from different fungal divisions can be achieved. Similar in vivo recombination approaches have been conducted by heterologous expression of the entire aurofusarin and bikaverin biosynthetic gene clusters from *Fusarium* spec. in *A. nidulans* [74]. Moreover, along with our studies on *A. niger*, an AMA1-based vector was constructed for an in vivo recombination of various DNA fragments (encoding the *uidA* color enzyme or fluorescent proteins) into the conidial pigment gene *wA* of *A. nidulans, i.e.* homologous to the *fwnA* locus in *A. niger,* highlighting the general suitability of this biobricks approach [75]. A third example comprises the successful integration of the calbistrin cluster using six DNA fragments in an engineered *Penicillium rubens* platform strain resulting in the production of decumbenones A–C [76]. Although the calbistrin cluster is derived from the highly related species *Penicillium decumbens*, no production of calbistrins A–C was observed in the heterologous host, indicating that host specific effects can affect the metabolite production.

Indeed, inter-division gene expression is not a simple task because altered codon usage, intron recognition, protein folding or the lack of accessory proteins sometimes impedes successful heterologous protein production [9, 74]. Hence, it is the more astonishing as the NRPS gene *calA* from an EDF was successfully expressed in the ascomycete *A. niger*. However, the detected calpinactam titers are 71 times lower compared to the native producer, which may be caused by undesired splicing events or unstable transcripts in the host. The targeted recombination resulted in a single integration in the *fwnA* gene locus whilst multi-copy integrations may yield higher metabolite titers as shown in the biotechnological production of other natural compounds [17, 77]. To bypass this limitation, the introduced URA blaster might be excised by 5-fluoroorotic acid treatment of the expression strain to perform a second round of transformation into another locus [78].

The described system is suitable for expression of long genes of Basidiomycetes. We successfully assembled the 8 kb *lpaA* gene from five DNA fragments in *A. niger* and confirmed the functionality by the production of various methyl-branched polyenes of different chain length (C_26_–C_32_) and ∆^7,8^ *cis/trans* stereochemistry, which is in accordance with observations made by the heterologous expression of *lpaA* in *Aspergilli* in a previous report [41]. Increasing the metabolic diversity by premature chain truncation is a well-recognized phenomenon of fungal polyketide synthesis [20, 79, 80]. The C4-methyl group as part of the 1-methyl-2-oxo-propylidene head is a common feature of many fungal linear polyenes [49–51]. We showed that the inactivation of the C-MT of LpaA is critical for chain elongation and completely abolished laetiporic acid production. As demonstrated for the iterative type I PKS LovB in the lovastatin biosynthesis of *A. terreus*, the highly regioselective and comparably fast C-MT reactions are essential for the full extension of the lovastatin nonaketide chain [81, 82]. Deconstruction studies of single domains of the PKS PksCT producing the highly (C2,C4,C6)-trimethylated pentaketide citrinin showed that in absence of the C-MT domain, the minimal PKS solely produced an unmethylated triketide pyrone [52]. Similar to our results on the LpaA C-MT, single mutations in the catalytic dyad of the C-MT of the PKS *MtaltA* in the alternapyrone biosynthetic pathway completely abolished product formation [83]. Since no intermediates are detectable in the *lpaA* C-MT mutant, C4-methylation occurs most likely at the thioester α-carbon following the first extension cycle and not on the fully elongated hexacosaketide.

In a second approach, we successfully integrated the 27.7 kb *calA* expression construct (including the *calA* gene and the required regulatory elements) into the genome of *A. niger.* The calpinactam synthetase CalA (712 kDa) is approximately one and a half the size of the largest heterologously NRPSs produced in *S. cerevisiae*, among them the α-l-aminoadipyl-l-cysteinyl-d-valine synthetase PcbAB (426 kDa), the fumiquinazoline F synthetase Afu6g12080 (438 kDa), the tryptoquialanine synthetase TqaA (450 kDa) and the aspyridone synthetase ApdA (498 kDa) [84–87]. Our work enables both an optimized calpinactam production by the original producer strain *M. alpina* and the biotechnologically tractable *A. niger* strain tJMW06. Although, further improvement is required to increase the yield of the compound, e.g. by codon-optimization [88], the described tool provides the first evidence for a functional heterologous expression of a natural product gene in full-length from an early diverging fungus. Calpinactam represents a promising alternative to conventional, often less effective antibiotics to treat tuberculosis [89]. The compound possesses an unusual C-terminal ε-caprolactam ring and resembles the structure of mycobactin, i.e. the hydroxamate siderophore of *M. tuberculosis,* and may interfere with the iron uptake system [53]. Inhibition of iron metabolism is a prospective and highly specific target for antimycobacterial drug development [90].

In our studies, no linear calpinactam peptides with hydrolyzed ε-caprolactam ring system were detected as side products—neither in the original nor in the transgenic producer. This suggests that the 7-membered azacycle is not spontaneously formed, but rely on the intrinsic activity of the releasing module of CalA. Caprolactam-containing metabolites—such as the nucleoside antibiotic capuramycins [91], bangamide A [92], the antifungal circinatin [93], the cytotoxin caprolactin A [94], terreazepine [39], the muscarinic acetylcholine receptor inhibitor nocardimicin [95] and the siderophore mycobactin [90]—are frequently isolated from microorganisms. The caprolactam structure in capuramycin originates from l-lysine and has been assigned to the action of the NRPS-like module CapU [91]. Future investigations on the 6th module of CalA might be of importance, as the current industrial production routes to ε-caprolactam, i.e. the monomeric precursor of the widely used synthetic fibers nylon-6 and PEBA2000 [96, 97], rely on expensive and energy-wasting classical chemical syntheses [98]. Alternative, sustainable production routes in mammalian cells [99], plants [98] or fungi are highly requested bio-based strategies.

## Conclusions

The heterologous expression of genes encoding megasynth(et)ases for natural products has long time been a very challenging task. The presented expression system allows integration and expression of long genes of interest of > 20 kb in a single step. The tool is suitable for genes of various biological sources, including higher and early diverging fungi, and provide the basis to tap yet unexplored secondary metabolite producers such as non-cultivatable fungi.

## Methods

### Organisms and culture conditions

*Aspergillus niger* strains ATNT, tLK01 (ATNT∆*akuB*), the *lpaA*-expressing strains tLK04 and tLK05, the *calA*-producing strain tJMW06 and the empty vector controls tLK06 and tLK07 were cultivated on *Aspergillus* Minimal Medium (AMM) agar plates supplemented with 2 mm l-glutamine [100]. To compensate uracil auxotrophy, 10 mm uridine (Carl Roth) was added for cultivation of the strains ATNT, tLK01 and tLK06. For tLK01 cultivation, 140 µg mL^−1^ hygromycin B (Carl Roth) was added. Plates were incubated at 30 °C for 4 d and conidia were harvested as previously described [41]. 100 mL AMM with 200 mm glucose and 20 mm l-glutamine or YPD (10 g L^−1^ yeast extract, 20 g L^−1^ soy peptone, 20 g L^−1^ d-glucose) were inoculated with a titer of 1 × 10^6^ *A. niger* conidia mL^−1^ to produce laetiporic acids at 30 °C for 72 h or to produce calpinactam at 25 °C for 72 h, respectively. Transgenic *A. niger* cultures were induced with 30 µg mL^−1^ doxycycline (Merck). *Mortierella alpina* ATCC32222 was cultivated on MEP agar plates (20 g L^−1^ malt extract, 3 g L^−1^ soy peptone, 20 g L^−1^ agar, pH 5.6) at 25 °C for 7 d. To produce calpinactam, 100 mL MEP liquid medium was inoculated by addition of three agar blocks (3 × 3 mm) and cultivated at 25 °C for 4 d. *Escherichia coli* XL1 blue were used for plasmid propagation and were cultivated in LB medium (5 g L^−1^ yeast extract, 10 g L^−1^ tryptone, 10 g L^−1^ NaCl) supplemented with 100 µg mL^−1^ carbenicillin (Roth), if required. Authentic standards for laetiporic acids were obtained from fruiting bodies of *Laetiporus sulphureus* JMRC:SF:012,599 as described [41]. All strains used in this study are listed in Additional file 3: Table S2.

### Strain construction

Details on the cloning procedures, strain transformation, used strains and oligonucleotides are given in the Additional file 1: Experimental procedures, Additional file 3: Table S2, Additional file 4: Table S3. In brief, the *akuB* deletion mutant (tLK01) was generated by replacing the entire gene (2651 bp) with the hygromycin B resistance cassette (*hph*) [45] from plasmid pLK03. To replace the spore pigment polyketide synthase gene *fwnA* by overexpression constructs, the pSMX2-derived [18] expression vector pPS01 [41] was expanded by an 1 kb upstream and 1 kb downstream flank of *fwnA* to yield plasmid pLK04. The plasmid served either as vector to ligate DNA sequences or as template for subsequent fusion PCRs. For heterologous expression of *lpaA* (*A. niger* strains tLK04 and tLK05) and *calA* (*A. niger* strain tJMW06) in the recipient strain tLK01, the five gene fragments of the laetiporic acid synthase gene (from *L. sulphureus*) and the calpinactam synthetase gene (from *M. alpina*) were amplified from the plasmid pPS03 [41] or the genomic DNA of *M. alpina* strain ATCC32222, respectively. In either case, the orotidin-5′-phosphate-decarboxylase gene, *pyrG*, from *Aspergillus nidulans* was used as selectable marker. The generated transformants were checked by PCR (Fig. 2, Additional file 8: Fig. S4, Additional file 12: Fig. S8). Deletion of *akuB* in *A. niger* tLK01 and *fwnA* in *A. niger* tLK04 as well as the full-length integration of *calA* in tJMW06 were additionally confirmed by Southern blot analysis with digoxigenin-labeled probes as described (Figures S1, Additional file 9: Fig. S5, Additional file 13: Fig. S9) [45].

### Expression and splicing analyses

To determine the expression of *lpaA* in *A. niger* (*lpaA*-expressing), tLK05 (*lpaA*^D1415A^-expressing) and tLK07 (null mutant), fungi where cultivated in 200 mL AMM supplemented with 200 mm d-glucose and 20 mm l-glutamine at 30 °C for 48 h. To determine the expression of *calA* and its processed splicing products *M. alpina* ATCC32222 and *A. niger* tJMW06 was cultivated in 200 mL MEP for 72 h or in 200 mL YPD amended by 30 µg mL^−1^ doxycycline for 36 h, respectively. Mycelium was harvested, ground under liquid nitrogen, and RNA was isolated using the SV Total RNA Isolation System (Promega). Residual gDNA was digested using Baseline-Zero DNase (Biozym). cDNA was synthesized using the anchored oligo(dT)_20_ primers and the RevertAid First Strand cDNA Synthesis Kit (Thermo Scientific). Semi-quantitative PCR was carried out with DreamTaq Green Polymerase (Thermo Scientific) and oligonucleotides listed in Additional file 4: Table S3 according to the manufacturer’s instructions. As internal standard, the housekeeping genes encoding actin B (*actB*; for *M. alpina*) or the glycerinaldehyde-3-phosphate-dehydrogenase (*gpdA*; for *A. niger*) was used.

### Extraction of laetiporic acids and calpinactam

Mycelia of *A. niger* tLK04 (*lpaA*-expressing), tLK05 (*lpaA*^D1415A^-expressing), tLK07 (empty vector control) and *L. sulphureus* fruiting bodies were used to extract laetiporic acids. Accordingly, mycelia of *A. niger* tJMW06 (*calA* expressing), tLK07 (empty vector control) and *M. alpina* were used to produce calpinactam. Mycelia were harvested by filtration, rinsed with water and finally lyophilized to entire dryness. Mycelia were ground to a fine powder using a mortar and pestle and subsequently extracted with 20 mL acetone per gram dry weight (laetiporic acids) or with 20 mL methanol per gram dry weight (calpinactam) at 150 rpm for 2 h. The solvent phase was decanted, filtered and evaporated under reduced pressure in a rotary evaporator. The residue was dissolved in 1 mL methanol and 5 µL thereof was subjected to UHPLC-MS analyses.

### Chemical standards

Authentic standards of laetiporic acids A_1_, A_2_, B_1_, B_2_, C_1_, and C_2_ were isolated from *L. sulphureus* fruiting bodies as described previously [41]. A commercial standard for calpinactam was obtained from Santa Cruz Biotechnology, Inc. (CAS 1205538-83-5).

### UHPLC-MS measurement

Extracts and standards were measured on an Agilent Infinity II 1290 system connected to an Agilent 6130 single quadrupole mass spectrometer. To detect laetiporic acids the following gradient was applied on a Zorbax Eclipse Plus C18 RRHD column (Agilent; 50 mm × 2.1 mm, 1.8 µm) at a flow rate of 1 mL min^−1^ at 30 °C using water + 0.1% formic acid (solvent A) and acetonitrile (solvent B): 0–3 min: 5–50% B; 3–7 min: 50–75% B; 7–8 min: 75–100% B. Signals were recorded at wavelengths λ = 210–600 nm by a diode array detector and UV profiles were extracted at λ = 450 nm. Additionally, extracted ion chromatograms were recorded by ESI–MS in positive ionization mode (*m/z* 421, 447, 473, 499 [*M* + H]^+^). Purified polyenes LA A_1_–LA C_2_ from *L. sulphureus* served as reference standard [41]. To detect calpinactam, the identical column and chromatograph were used and the following gradient were applied: 0–4 min: 5–72% B, 4–4.5 min: 72–95% B, 4.5–5 min: 95% B. Since calpinactam is hardly chromophore, mass signals were recorded in positive ionization mode and detection of calpinactam was based on selected ion monitoring (SIM) signals of *m/z* 768 [*M* + H]^+^. For quantifications, a calibration curve was recorded with a commercial calpinactam standard (Santa Cruz Biotechnology, Inc.) in concentrations ranging from 0.488 to 125 µg mL^−1^.

### High resolution mass spectrometry

HR-MS and HR-MS/MS spectra of identified compounds and the respective standards were additionally recorded on a Q Exactive Plus mass spectrometer (Thermo Scientific) as described for polyenes [41] and peptides [57].

## Supplementary Information

**Additional file 1. **Experimental procedures.

**Additional file 2: Table S1.** Calculation of the frequency of homologous recombination in *A. niger *ATNT and tLK01 transformed with the *fwnA* deletion construct. *T1-T3 indicate the number of transformants per 500 ng DNA from three independent transformations.

**Additional file 3: Table S2.** Organisms used in this study.

**Additional file 4: Table S3.** Oligonucleotides used in this study.

**Additional file 5: Figure S1.** Southern Blot analysis for determination of the *akuB* deletion strain tLK01. **A. **Schematic representation of the genomic *akuB* locus of the strain ATNT and tLK01 (ATNT∆*akuB*) with its respective *EcoR*V (upper panel) and *Hind*III restriction sites (lower panel). **B. **Southern Blot analysis of the *A. niger* parental strain ATNT and the *akuB* deletion strain tLK01**. **Genomic DNA was digested with *EcoR*V or *Hind*III. A digoxigenin-labeled probe was generated with oMG482/oMG483 to hybridize with the *akuB *upstream sequence and signals were detected with CDPstar (Roche Diagnostics).

**Additional file 6: Figure S2.** Plasmid maps of the expression vectors. The plasmids pLK04 (**A**), pLK05 (**B**), pMG56 (**C**) and pMG58 (**D**) are based on the pSMX2-URA plasmid [1]. The gene fragments are: *bla*; β-lactamase (confers ampicillin resistance); *calA1/5*; 1 kb of the 5′or 3′ end of the *calA* gene; *fwnA*up and* fwnA*down, 1 kb up- and downstream the *A. niger fwnA* polyketide synthase gene; URA-blaster (dark blue, contains several genes; see below); P*terA*, promoter of the *terA* terrein polyketide synthase gene of *Aspergillus terreus*; Tag, encodes the hexahistidin tag (and includes an *SpeI* and *PacI* site for insertion of the GOI); T*trpC*, terminator of the *trpC *anthranilatsynthase component 2 gene of *A. terreus*; rep origin, origin of plasmid replication. The URA-blaster contains: P*pyrG*, promoter of the *pyrG* gene from *Aspergillus nidulans*; *pyrG*, orotidine 5′-phosphate decarboxylase gene from *A. nidulans* (confers uracil prototrophy); T*pyrG*, terminator of the *pyrG* gene from *A. nidulans*; and two *prpB *flanks that encode the methylcitrate synthase gene from *Escherichia coli* that facilitate a subsequent removal of the URA blaster cassette from the *A. niger* genome via a homologous recombination event (counter selection) by addition of 5-fluoroorotic acid [2].

**Additional file 7: Figure S3.** Photographs of *A. niger *ATNT and tLK01 transformed with the *fwnA* deletion construct. Both pigmented and non-pigmented transformants have been detected in both experiments, but frequency of the homologous recombination into the *fwnA* locus is significantly higher in tLK01. For calculation of frequency of recombination see Additional file 2: Table S1.

**Additional file 8: Figure S4.** Determination of the homologous integration of the P*terA*:T*trpC *construct into the *fwnA* locus of recipient strains ATNT and tLK01. **A. **Schematic representation of the genomic *fwnA* locus of the parental strain ATNT and tLK01 (ATNT∆*akuB*) (upper lane) and the deletion mutants ATNT∆*fwnA* (tLK06) and tLK07 (ATNT∆*akuB*∆*fwnA*). **B. **Agarose gel of two diagnostic PCRs targeting the *fwnA* gene (upper lane) and the P*terA* promoter (lower lane). In contrast to the parental strains ATNT and tLK01, the non-pigmented ∆*fwnA* mutants (tLK06 and tLK07) lack the signal of the *fwnA* gene (upper panel). In *lieu *thereof, the homologous integration of P*terA*:T*trpC* could be determined in the mutants (lower panel).

**Additional file 9: Figure S5.** Southern Blot analysis for determination of the *fwnA* deletion and *lpaA* overexpression in *A.* *niger* strains tLK04. **A. **Schematic representation of the genomic *fwnA* locus in the strain tLK01 (ATNT∆*akuB*) and the ∆*fwnA*::*lpaA* overexpression strain tLK04 with its respective *Sac*II restriction sites. **B. **Southern Blot analysis of the *A. niger* parental strain tLK01 and five ∆*fwnA*::*lpaA* overexpression strains tLK04**. **Genomic DNA was digested with *Sac*II. A digoxigenin-labeled probe was generated with oMG504/oMG505 to hybridize with the *fwnA *downstream sequence and signals were detected with CDPstar (Roche Diagnostics). Strains used for subsequent metabolic analysis are highlighted in green.

**Additional file 10: Figure S6.** HR-MS and MS/MS spectra of laetiporic acids A_1_-D_2_ detectable in *A.* *niger* tLK04. High resolution MS/MS fragmentation of laetiporic acids A_1_/A_2_ (**A**), B_1_/B_2_ (**B**), C_1_/C_2_ (**C**) and D_1_/D_2_ (**D**) produced by *A*. *niger *tLK04. Indicated fragments are identical to the literature [1].

**Additional file 11: Figure S7.** Expression of *lpaA* in the transgenic *A. niger* tLK07 (null mutant), tLK04 (*lpaA* expressing) and tLK05 (*lpaA*^D1415A^ expressing). Expression was profiled by semi-quantitative PCR on the laetiporic acid synthase gene (*lpaA*) and referenced to the expression of the housekeeping gene encoding the glyceraldehyde-3-phosphate dehydrogenase (*gpdA*). RNA was isolated and cDNA was synthesized after cultivation for 36 h in inducing AMM (with doxycycline) at 30°C and 180 rpm. The genomic DNA (gDNA) of tLK07 or the *lpaA*-encoding plasmid pPS03 served as positive controls for *gpdA* and *lpaA* amplification, respectively.

**Additional file 12: Figure S8.** Integration of *calA* into the *fwnA* locus in *A.niger* tJMW06. **A. **Genomic locus of *fwnA* during recombination of the five *calA* DNA fragments in *A. niger *pJMW06.** B.** PCR amplification of four adjacent DNA fragment pairs was carried out using genomic DNA as templates. The *A. niger *parental strain tLK01 and the null mutant strain tLK07 (empty vector) served as negative controls. Three individual *calA*-expressing transformants tJMW06 #3, #13 and #26 showed the expected amplicon sizes of the recombined DNA fragments.

**Additional file 13: Figure S9.** Southern Blot analysis for determination of the full-length *calA* integration into the genome of *A. niger *strain tJMW06. **A**. Schematic representation of the genomic *fwnA* locus in the strain tLK01 (ATNT∆*akuB*), the∆*fwnA*::*calA* overexpression locus of strain tJMW06.3 and the native *calA* locus of the *calA* gene donor strain *M. alpina* ATCC32222. **B. **Southern Blot analysis of the *A. niger* parental strain tLK01, the ∆*fwnA*::*calA* overexpression strain tJMW06 and *M. alpina* ATCC32222**. **Genomic DNA was double-digested with *Sma*I/*Dra*I. A digoxigenin-labeled probe was generated with oMG569/oMG548 to hybridize with the *fwnA *downstream sequence and signals were detected with CDPstar (Roche Diagnostics). Full-length *calA* integration was determined for *A. niger* tJMW06 and directly reflects the size of the *calA* gene fragment in the gene donor strain *M. alpina* ATCC32222.

**Additional file 14: Figure S10.** Temperature dependent production of calpinactam in *A. niger *tJMW06 and *M. alpina* ATCC32222*.* Calpinactam production (bars) and total fungal dry weight (boxes) are indicated for several cultivation conditions. The transformant *A. niger* tJMW06 was cultivated in YPD with 30 µg mL^1^ doxycycline as inducer at 20, 25 and 30°C for 3 days. The *calA* gene donor strain *M. alpina* ATCC32222 was cultivated in MEP (25°C) for 4 days. Production rate in *A. niger* tJMW06 is optimal at 25 °C, which is the growth optimum for *M. alpina.* Experiments were carried in triplicate.

## Data Availability

The sequence of the *calA* gene from *M. alpina* ATCC32222 is deposited under the GenBank accession number OP959498. The fungal strains are available upon request from the American Type Culture Collection (ATCC) or the Jena Microbial Resource Collection (JMRC) as listed in Additional file 3: Table S2.
