# Supplementary material for: A genetic tool to express long fungal biosynthetic genes
Source: Fungal Biol Biotechnol. 2023 Feb 1;10:4. doi: 10.1186/s40694-023-00152-3 (PMC9893682; doi:10.1186/s40694-023-00152-3)
Supplement: Supplementary file 1 — Additional file 1. Experimental procedures. [file 40694_2023_152_MOESM1_ESM.pdf]

A Genetic Tool to Express Long Fungal Biosynthetic Genes

Leo Kirchgaessner,[1,2,3] Jacob M. Wurlitzer,[1,2] Paula S. Seibold, [1,2] Malik Rakhmanov,[1,2] and Markus Gressler[1,2]

[1] Friedrich Schiller University Jena, Institute of Pharmacy, Department Pharmaceutical Microbiology, Winzerlaer Strasse 2, 07745 Jena, Germany

[2] Leibniz Institute for Natural Product Research and Infection Biology – Hans Knöll Institute, Department Pharmaceutical Microbiology, Winzerlaer Strasse 2, 07745 Jena, Germany

[3] Ernst Abbe University of Applied Sciences Jena, Faculty Medical Technology and Biotechnology, Carl-Zeiss-Promenade 2, 07745 Jena, Germany

Supporting information

Experimental procedures 2

A. Cloning and transformation procedures 2

B. Genetic characterization of the *lpaA* and *calA* expressing *A. niger* mutants 5

References 6

Experimental procedures

A. Cloning and transformation procedures

General remarks. Polymerase chain reactions (PCRs) were carried out with the HotStart Phusion polymerase (NEB; for < 8 kb fragments), the Phire Hot Start II DNA Polymerase (Thermo Scientific; for > 8 kb fragments) or the DreamTaq Green DNA-Polymerase (Thermo Scientific; for diagnostic PCRs for <2 kb fragments) according to the manufacturer´s protocol. Oligonucleotides are listed in Table S3**.** Plasmid propagation was carried out in *Escherichia coli* XL1-blue. *A. niger* mutants were generated by PEG-mediated transformation of protoplasts by an established protocol [1]. Organisms used and generated in this study are listed in Table S2. Cultivation media were LB medium (5 g L^{-1} yeast extract, 10 g L^{-1} tryptone, 10 g L^{-1} NaCl), YPD medium (10 g L^{-1} yeast extract, 20 g L^{-1} soy peptone, 20 g L^{-1} D-glucose), MEP medium (20 g L^{-1} malt extract, 3 g L^{-1} soy peptone, pH 5.6), *Aspergillus* minimal medium (AMM, https://www.fgsc.net/methods/anidmed.html) and AMM supplemented with 200 mM D-glucose and 20 mM L-glutamine. If required, 140 µg mL^{-1} hygromycin B (Roth), 100 µg mL^{-1} carbenicillin (Roth), 30 µg mL^{-1} doxycycline (Merck) or 10 mM uridine (Roth) was used for antibiotic selection, *tetON* promoter induction or auxotrophic complementation.

Generation of the *akuB* deletion in *A. niger* ATNT. The 5´and 3´flanking regions (1 kb each) of the *akuB* gene were amplified by PCR using the Phusion Polymerase and the oligonucleotide couples oMG482/oMG483 (*akuB*up) and oMG484/oMG485 (*akuB*dn), respectively. Both amplicons were fused by PCR [1] and ligated into the plasmid pJET1.2. The fusion fragment was excised by *Kpn*I and ligated into an analogously digested pUC19 vector resulting in pLK02. The hygromycin B resistance (*hph*) cassette (2.9 kb) was obtained by *Not*I-digest of *hph*-pCRIV [1] and was inserted into the *Not*I-linearized pLK02 to give plasmid pLK03. The deletion cassette (*akuB*up-*hph*-*akuB*dn, 5 µg) was excised by *Kpn*I, gel-purified and used for PEG-mediated transformation protoplast of *A. niger* ATNT [2, 3]. Single transformants were selected on AMM plates with 140 µg/ml hygromycin B. Genomic DNA of the parental strain and the *akuB* deletion strain (tLK01) was digested separately with *Eco*RV and *Hind*III

and Southern Blot analysis was conducted using a digoxygenin-labelled probe amplified from pLK03 with oligonucleotides oMG482/oMG483 (Figure S1).

Generation of the expression plasmid pLK04. The upstream (*fwnA*up, 1007 bp) and the downstream fragment (*fwnA*dn, 1037 bp) of the *fwnA* gene were amplified using oligonucleotide pairs oMG501/oMG502 and oMG504/oMG505, respectively. An additional fragment (spanning the inducible *Aspergillus terreus terA* promoter, the *A. terreus trpC* terminator and the *Aspergillus fumigatus pyrG* gene) was amplified from plasmid pPS01 [4] using oMG370 and oMG109. The three fragments were fused with a *Pst*I-linearized pUC19 vector using the NEBuilder Assembly Tool (NEB). The final plasmid pLK04 is a bifunctional vector for deletion of the spore pigment PKS gene *fwnA* and simultaneous overexpression of genes of interest (GOI) in *Aspergillus niger* ATNT (Figure S2). The GOI can be integrated via *Pac*I or *Spe*I digest resulting in either N-terminal or C-terminal His_6-tagged proteins.

Generation of the null mutants *A. niger* tLK06 and tLK07 and determination of the frequency of recombination. To test the frequency of homologous recombination events in tLK01 in comparison to its parental strain ATNT, both strains were transformed with 500 ng of two empty vector fragments targeting the *fwnA* locus (primer pairs oMG501/oMG116 and oMG370/oMG505 priming pLK04; 2,054 bp and 5,827 bp, respectively). The experiment was carried out in triplicate. All black-colored and pigment-less colonies were counted for each transformation (tLK06 and tLK07) and frequency of homologous recombination was calculated as ratio of (fawn colonies) / (black + fawn colonies). Three white transformants from each transformation were checked for *fwnA* deletion by PCR using the oligonucleotides oMG539/oMG541 and oMG539 /oMG267.

Generation of the *lpaA* expression plasmid pLK05. The first 1027 bp of *lpaA* (*lpaA*1) and the terminal 1014 bp of *lpaA* (*lpaA*5) were amplified from pPS03 using oligonucleotides oMG527/oMG528 and oMG529/oMG530. Both fragments were fused by PCR as described [1] and ligated into the *Pac*I-digested expression vector pLK04. The obtained plasmid pLK05 served as template for various amplifications of *lpaA* fragments (see below).

Generation of the *lpaA*-expressing mutant *A. niger* tLK04 and the *lpaA*^{D1415A}-expressing mutant *A. niger* tLK05. The recipient strain tLK01 was transformed by PEG-mediated transformation using five DNA fragments. Fragment 1 (2,792 bp) spanned *fwnA*up:P*terA*:*lpaA*1 and was amplified from pLK05 using oMG501/oMG532. Fragments 2 (2,897 bp), 3 (3,966 bp), and 4 (3,669 bp) spanning overlapping regions of the *lpaA* gene were amplified from pPS03 using oCL46/oCL49, oCL34/oCL51, and oCL42/oMG530. For tLK05, fragment 3 was replaced by a fusion PCR construct amplified by PCR fusion of fragments 3a (2,437 bp) and 3b (1,581 bp), each amplified by oPS30/oPS45 and oPS46/oPS31. Finally, fragment 5 (5,971 bp) was generated from pLK05 using oMG531/oMG505 and spanned *lpaA*5*:*T*trpC:pyrG:fwnA*dn. All fragments (400-600 ng each) were mixed in an equimolar ratio in a final volume of 20 µl and were used for PEG-mediated transformation of the recipient strain tLK01. The resulting transformants (tLK04 and tLK05) were primarily screened by loss of conidial pigmentation and the genotype were subsequently determined by PCR (oMG370/oMG116) (Figure 2) and Southern Blot (Figure S5).

Generation of the *calA* expression plasmids pMG56 and pMG58. The first 846 bp of *calA* (*calA*1) and were amplified from genomic DNA from *M. alpina* ATCC32222 using oligonucleotides oMG569/oMG548. *cal*A1 was fused with the *Pac*I-digested expression vector pLK04 using the NEBuilder Assembly Tool (NEB) to obtain plasmid pMG56. The terminal 1026 bp of *calA* (*calA*5) was amplified using oligonucleotides oMG546/oMG547, subcloned into the pJET1.2 vector and finally ligated by *Mfe*I/ *Pac*I excision into a similar digested pLK04 to result in pMG58. Both vectors served as a template for various amplifications of *calA* fragments (see below).

Generation of the *calA*-expressing mutant *A. niger* tJMW06. The recipient strain tLK01 was transformed by PEG-mediated transformation using five DNA fragments. Fragment 1 spanned *fwnA*up:P*terA*:*calA*1 and was amplified from pMG56 using oMG501/oMG548. Fragments 2 (7,497 bp), 3 (7,607 bp), and 4 (6,875 bp) spanning overlapping regions of the *calA* gene were amplified from genomic DNA from *M. alpina* using oMG572/oMG507, oMG570/oMG509, and oMG510/oMG511. Finally, fragment 5 was amplified from pMG58 using oMG505/oMG547 and spanned

*calA*5:T*trpC:pyrG:fwnA*dn. All fragments (250 ng/ kb each) were mixed in an equimolar ratio in a final volume of 20 µl and were used for PEG-mediated protoplast transformation of the recipient strain tLK01. The genotype of the resulting transformants (tJMW06) was subsequently analyzed by PCR (Figure S8) and Southern Blot (Figure S9).

B. Genetic characterization of the *lpaA* and *calA* expressing *A. niger* mutants

Isolation of genomic DNA. *A. niger* strains or *M. alpina* were cultivated in 50 ml MEP for 36 h at 25°C. Mycelium was ground under liquid nitrogen and suspended in LETS buffer (100 mM lithium chloride, 20 mM EDTA, 10 mM TRIS, 0.5 % SDS, pH 8.0) and incubated with 25 µg ml^{-1} Monarch RNase A (NEB) at 65°C. After addition of 25 µg ml^{-1} Proteinase K (Merck) and a second incubation at 65°C, cell debris was removed by brief centrifugation at 20,000 × g. The cell-free supernatant was extracted three times with phenol/ chloroform/ isoamyl alcohol (25:24:1) (Carl Roth) and the organic phase was discarded every time. The DNA in the aqueous phase was precipitated by addition of the equal amount of ice-cold isopropanol. After centrifugation (4°C, 20,000 × g, 10 min), the DNA pellet was washed with 70% ethanol and dried at 37°C for 3 minutes. Finally, the gDNA was solved in 10 mM TRIS buffer (pH 8.0) and stored at 4°C.

Diagnostic PCR of the transformants. To estimate the integration of expression constructs, 200 ng genomic DNA of *A. niger* ATNT, tLK01, tLK04, tLK05, tLK06, tLK07 and tJMW06 and *M. alpin*a ATCC32222 served as template for PCR's using the Phire Hot Start II DNA Polymerase (for > 8kb fragments), HotStart Phusion polymerase (for 2-8 kb fragments), DreamTaq Green DNA Polymerase (for <2 kb fragments) according to the manufacturer´s protocols. The oligonucleotides used for each experiment are listed in Table S3 and are indicated in the figure legends of Figures 2, 5, S4, S7 and S8.

Southern Blot analysis. To verify the correct and full-length integration, Southern blot analysis were carried out. To determine the *akuB* deletion, genomic DNAs of ATNT and tLK01 were digested with *Hind*III or *Nco*I. To determine the *fwnA* deletion, the genomic DNA of tLK01 and tLK04 was digested

with *Sac*II. To determine the full-length integration of *calA* deletion, the genomic DNA of tLK01 and tLK04 was digested with *Sma*I/*Dra*I. Digested DNA fragments were electrophoreticly separated on a 0.7% agarose gel. Gels were treated with depurination solution (0.25 M HCl), denaturation solution (0.5 M NaOH, 1.5 M NaCl) and neutralization solution (1.5 M NaCl, 0.5 M TRIS, pH 7.5). Capillary blot onto an Amersham Hybond-N nylon membrane (VWR) was conducted in 20 x SSC buffer (0.3 M sodium citrate, 3 M NaCl) for 3 hours. Subsequently, DNA was cross-linked to the membrane by UV radiation. Hybridization, DNA labeling and detection was performed using the DIG-High Prime DNA Labeling and Detection Starter Kit II (Roche) and the Anti-Dig AP Fab fragment (Roche) according to manufacturer´s instructions. Probes were amplified from plasmid DNA (pLK03, pLK04 and pMG56) using DIG-11-UTP (Jena Bioscience) nucleotides and oligonucleotide pairs oMG482/483, oM504/505 and oMG569/548 for probes targeting *akuB*up, *fwnA*dn and *calA*, respectively.

References

1. Gressler M, Zaehle C, Scherlach K, Hertweck C, Brock M: **Multifactorial induction of an orphan PKS-NRPS gene cluster in *Aspergillus terreus***. *Chem Biol* 2011, **18**(2):198-209.
2. Geib E, Brock M: **ATNT: an enhanced system for expression of polycistronic secondary metabolite gene clusters in *Aspergillus niger***. *Fungal Biol Biotechnol* 2017, **4**(1):e13.
3. Geib E, Baldeweg F, Doerfer M, Nett M, Brock M: **Cross-Chemistry Leads to Product Diversity from Atromentin Synthetases in Aspergilli from Section Nigri**. *Cell Chem Biol* 2019, **26**(2):223-234 e226.
4. Seibold PS, Lenz C, Gressler M, Hoffmeister D: **The *Laetiporus* polyketide synthase LpaA produces a series of antifungal polyenes**. *J Antibiot (Tokyo)* 2020, **73**(10):711-720.
