# Supplementary material for: A genetic tool to express long fungal biosynthetic genes
Source: Fungal Biol Biotechnol. 2023 Feb 1;10:4. doi: 10.1186/s40694-023-00152-3 (PMC9893682; doi:10.1186/s40694-023-00152-3)
Supplement: Supplementary file 2 — Additional file 2: Table S1. Calculation of the frequency of homologous recombination in A. niger ATNT and tLK01 transformed with the fwnA deletion construct. *T1-T3 indicate the number of transformants per 500 ng DNA from three independent transformations. [file 40694_2023_152_MOESM2_ESM.pdf]

Table S1. Calculation of the frequency of homologous recombination in *A. niger* ATNT and tLK01 transformed with the *fwnA* deletion construct. *T1-T3 indicate the number of transformants per 500 ng DNA from three independent transformations.

parental strain	phenotype	T 1*	T 2*	T 3*	sum	frequency (HR)
**ATNT**	pigmented (ectopic)	8	9	10	27	
	non-pigmented (Δ*fwnA*)	2	1	2	5	**15%**
**tLK01**	pigmented (ectopic)	1	3	3	7	
	non-pigmented (Δ*fwnA*)	9	10	9	28	**80%**
