# Supplementary material for: A genetic tool to express long fungal biosynthetic genes
Source: Fungal Biol Biotechnol. 2023 Feb 1;10:4. doi: 10.1186/s40694-023-00152-3 (PMC9893682; doi:10.1186/s40694-023-00152-3)
Supplement: Supplementary file 3 — Additional file 3: Table S2. Organisms used in this study. [file 40694_2023_152_MOESM3_ESM.pdf]

Table S2. Organisms used in this study.

strain	genotype	remarks	reference
Aspergillus niger ATNTΔpyrG	TetOn:*terR_ble*; *pyrG*::*ptrA*	expression platform	[1]
Aspergillus niger tLK01	TetOn:*terR_ble*; *pyrG*::*ptrA; ΔakuB::hph*	expression platform for homologous integration	this study
Aspergillus niger tLK04	TetOn:*terR_ble*; *pyrG*::*ptrA; ΔakuB::hph; ΔfwnA::PterA:His6:lpaA_pyrG*	laetiporic acid production	this study
Aspergillus niger tLK05	TetOn:*terR_ble*; *pyrG*::*ptrA; ΔakuB::hph; ΔfwnA::PterA:His6:lpaA*^{D1415A}*_pyrG*	no laetiporic acid production	this study
Aspergillus niger tLK06	TetOn:*terR_ble*; *pyrG*::*ptrA; ΔfwnA::PterA:His6_pyrG*	-	this study
Aspergillus niger tLK07	TetOn:*terR_ble*; *pyrG*::*ptrA; ΔakuB::hph; ΔfwnA::PterA:His6_pyrG*	empty vector control	this study
Aspergillus niger tJMW06	TetOn:*terR_ble*; *pyrG*::*ptrA; ΔakuB::hph; ΔfwnA::PterA:His6:calA_pyrG*	calpinactam production	this study
Mortierella alpina ATCC32222	wildtype	native calpinactam producer	ATCC
Laetiporus sulphureus JMRC:SF:012599	wildtype	native laetiporic acids producer	JMRC [2]

References

1. Geib E, Brock M: **ATNT: an enhanced system for expression of polycistronic secondary metabolite gene clusters in *Aspergillus niger***. *Fungal Biol Biotechnol* 2017, **4**(1):e13
2. Seibold PS, Lenz C, Gressler M, Hoffmeister D: **The *Laetiporus* polyketide synthase LpaA produces a series of antifungal polyenes**. *J Antibiot (Tokyo)* 2020, **73**(10):711-720.
