# Supplementary material for: A genetic tool to express long fungal biosynthetic genes
Source: Fungal Biol Biotechnol. 2023 Feb 1;10:4. doi: 10.1186/s40694-023-00152-3 (PMC9893682; doi:10.1186/s40694-023-00152-3)
Supplement: Supplementary file 4 — Additional file 4: Table S3. Oligonucleotides used in this study. [file 40694_2023_152_MOESM4_ESM.pdf]

Table S3. Oligonucleotides used in this study.

name	sequence 5'-3'	target	purpose/restriction site
oMG482	GAATTCGAGCTCGGTACCTCTGACAGAGAATGCGGAGG	*A. niger akuB*	*akuB* deletion/ Southern Blot probe "*akuB*up"
oMG483	CGGTCTGCGCGGCCGCTTCCAGTATGAATGACTGAGATTATTGATG	*A. niger akuB*	*akuB* deletion/ Southern Blot probe "*akuB*up"
oMG484	CATACTGGAAGCGGCCGCGCAGACCGTTATTAATCCCTATTAG	*A. niger akuB*	*akuB* deletion
oMG485	GGATCCCCGGGTACCCGCAGCGACAGCATGAGAG	*A. niger akuB*	*akuB* deletion
oMG501	CCTCTAGAGTCGACCTGCAGGAGTTTCGGAAGTTGAATAAGC	*A. niger fwnA*	*fwnA* deletion
oMG502	ACAATATCAGAGAGAGGATCGTTTGCGGAAGATGTCTATTGATC	*A. niger fwnA*	*fwnA* deletion
oMG504	TGATGGTGCCAACAATCTGCATTGTTATGCATTACGCCTTCCTCC	*A. niger fwnA*	*fwnA* deletion / Southern Blot probe "*fwn*Adn"
oMG505	CCAAGCTTGCATGCCTGCAGCCTGTTCATACTACTCCCTGCC	*A. niger fwnA*	*fwnA* deletion / Southern Blot probe "*fwn*Adn"
oMG370	GATCCTCTCTCTGATATTGTCG	*A. terreus* P*terA*	amplification of P*terA*::T*trpC*::*pyrG* expression cassette / full-length integration of *lpaA* and *lpaA*^{D1415A}
oMG109	GAGCGGATAACAATTTCACACAGG	pUC19	amplification of P*terA*::T*trpC*::*pyrG* expression cassette
oMG527	ATCACCATCACCATTTAATTAACATGGCAGTGAGGCCGCCCAGC	*L. sulphureus lpaA*	amplification of *lpaA*/ construction of pLK05
oMG528	ACAGTCATGTATGAGCTGAGGTTGGCCTCGAGGGAGTCTC	*L. sulphureus lpaA*	amplification of *lpaA*/ construction of pLK05
oMG529	GAGACTCCCTCGAGGCCAACCTCAGCTCATACATGACTGTGG	*L. sulphureus lpaA*	amplification of *lpaA*/ construction of pLK05
oMG530	ATTGAAATCACTGCTGCTAGTTAATTAATCATGCCACAATGCCGTTCAATATC	*L. sulphureus lpaA*	amplification of *lpaA*/ construction of pLK05
oMG532	GTTGGCCTCGAGGGAGTCTC	*L. sulphureus lpaA*	amplification of *lpaA1*
oCL49	GGTAACGCCAGGGAACTCGAGGGCCATCTCG	*L. sulphureus lpaA*	amplification of *lpaA2*

oCL34	GGTATCAAGCCAAACGCTATGC	*L. sulphureus lpaA*	amplification of *lpaA3*
oCL51	GGTAGAGAAGATGGGCACGGCTGGGGAAGAGC	*L. sulphureus lpaA*	amplification of *lpaA3*
oCL42	GGATGGATTTCATCTTCGG	*L. sulphureus lpaA*	amplification of *lpaA4*
oMG531	CTCAGCTCATACATGACTGTGG	*L. sulphureus lpaA*	amplification of *lpaA5*
oCL46	ATGGCAGTGAGGCCGCCCAGCTAC	*L. sulphureus lpaA*	amplification of *lpaA* (full length) - intergar
oCL47	TCATGCCACAATGCCGTTCAATATCTC	*L. sulphureus lpaA*	amplification of *lpaA* (full length)
oPS30	GCTCTTCCCACCACAGAACA	*L. sulphureus lpaA*	amplification of *lpaA3*^{D1415A}
oPS45	TCGATATGGCCGTGCTGACATAATC	*L. sulphureus lpaA*	amplification of *lpaA3*^{D1415A}
oPS46	GATTATGTCAGCACGGCCATATCGA	*L. sulphureus lpaA*	amplification of *lpaA3*^{D1415A}
oPS31	CATCAGGGTTCAATGGCGTC	*L. sulphureus lpaA*	amplification of *lpaA3*^{D1415A}
oMG116	GAGATGTGGTAGACGATTGATCC	*A. terreus* T*trpC*	integration of *calA /*
			full-length integration of *lpaA* and *lpaA*^{D1415A}
oMG539	CAGCATGTTGGTTATATATTCGAGC	*A. niger fwnAup*	control homologous integration *fwnA* locus
oMG541	CTCCGAAATGCCTCTTTAGTTCG	*A. niger fwnAdown*	control homologous integration *fwnA* locus
oMG267	CATGGTGCTGTGATGAGAAG	pSMX2	control homologous integration *fwnA* locus
oMG569	CTTCTCATCACAGCACCATGACTAGTATGACCCGGCAGCCTTCAGAG	*M.alpina calA*	amplification of *calA1 /* Southern Blot probe *"calA"*
oMG548	CAACTGATGGATGCAGCGATC	*M.alpina calA*	amplification of *calA1 /* Southern Blot probe *"calA"*
oMG572	ATGACCCGGCAGCCTTCAG	*M.alpina calA*	amplification of *calA2*
oMG507	GCCTTCTCTACTTGATCCTCG	*M.alpina calA*	amplification of *calA2*
oMG570	GCTACGACCCCACTTGCTCC	*M.alpina calA*	amplification of *calA3*

oMG509	GAGTTCTCTTGGTCTGGTCTTCG	*M.alpina calA*	amplification of *calA3*
oMG510	GTGACCACTCTACGCTCGAGG	*M.alpina calA*	amplification of *calA4*
oMG511	CTACATCAGGTCCTCAATCCTTGC	*M.alpina calA*	amplification of *calA4*
oMG547	CCCGATAGCGCGTCTTTTGC	*M.alpina calA*	amplification of *calA5*
oMG546	TGAAATCACTGCTGCTAGTTAATTAACTACATCAGGTCCTCAATCCTTGC	*M.alpina calA*	amplification of *calA5*
oMG582	ATGTCTTCGAAGTCCCACCTC	*M.alpina calA*	verification of integration of *calA*
oMG25	GTATGTGCAAGGCCGGTTTCG	*M.alpina actB*	expression analysis (housekeeping gene)
oMG26	GTGACACCATCGCCAGAATCG	*M.alpina actB*	expression analysis (housekeeping gene)
oMG429	GCTGTCGGCAAGGTCATCC	*A. niger gpdA*	expression analysis (housekeeping gene)
oMG430	CTTGACGAAGTTGGAGTTAAGG	*A.niger gpdA*	expression analysis (housekeeping gene)
oMG659	AACAGAATCTGGATCCTGCG	*L. sulphureus lpaA*	expression analysis (*lpaA* expression)
oMG660	GCCATATGAGAAACAGAGCG	*L. sulphureus lpaA*	expression analysis (*lpaA* expression)
oMR35	CTCAGAACAAATTTGGACCGC	*A. niger fwnAup*	integration control of *calA*
oJMW251	GCTGCAGGACAAGGAGGATG	*M.alpina calA*	splicing of *calA* (intron 1)
oJMW252	GATGGTGGTCGAGAGCTTTC	*M.alpina calA*	splicing of *calA* (intron 1)/ integration of *calA*
oJMW253	GCTTCGCCCGATATGCCAG	*M.alpina calA*	splicing of *calA* (intron 2) / integration of *calA*
oJMW254	CTTGCTCGCCTCCACAGAC	*M.alpina calA*	splicing of *calA* (intron 2)
oJMW255	GACGACCGATGCGTTCACC	*M.alpina calA*	splicing of *calA* (intron 3)
oJMW256	CTCCGTATGACAGCACAGCC	*M.alpina calA*	splicing of *calA* (intron 3) / integration of *calA*
oJMW257	GATCGGTCTGTTGGTGAGGC	*M.alpina calA*	splicing of *calA* (intron 4) / integration of *calA*

oJMW258	CTCGAGCACGGCTGAGTAG	*M.alpina calA*	splicing of *calA* (intron 4)
oJMW259	GGAACAAGACCGAGGCTTCC	*M.alpina calA*	splicing of *calA* (intron 5)
oJMW260	TGGATCAGATGCTCGGGACC	*M.alpina calA*	splicing of *calA* (intron 5) / integration of *calA*
oJMW261	CCCGCTTCAGTCACTTTCAGTC	*M.alpina calA*	splicing of *calA* (intron 6) / integration of *calA*
oJMW262	CCGTGTAAGCCTCAATCAATCC	*M.alpina calA*	splicing of *calA* (intron 6)
oJMW263	CATCTCCGACAGTGGTGCC	*M.alpina calA*	amplification of *calA* probe
oJMW264	GGAAGTCCAGTCGATCCGG	*M.alpina calA*	amplification of *calA* probe
