# Supplementary material for: A genetic tool to express long fungal biosynthetic genes
Source: Fungal Biol Biotechnol. 2023 Feb 1;10:4. doi: 10.1186/s40694-023-00152-3 (PMC9893682; doi:10.1186/s40694-023-00152-3)
Supplement: Supplementary file 5 — Additional file 5: Figure S1. Southern Blot analysis for determination of the akuB deletion strain tLK01. A. Schematic representation of the genomic akuB locus of the strain ATNT and tLK01 (ATNT∆akuB) with its respective EcoRV (upper panel) and HindIII restriction sites (lower panel). B. Southern Blot analysis of the A. niger parental strain ATNT and the akuB deletion strain tLK01. Genomic DNA was digested with EcoRV or HindIII. A digoxigenin-labeled probe was generated with oMG482/oMG483 to hybridize with the akuB upstream sequence and signals were detected with CDPstar (Roche Diagnostics). [file 40694_2023_152_MOESM5_ESM.pdf]

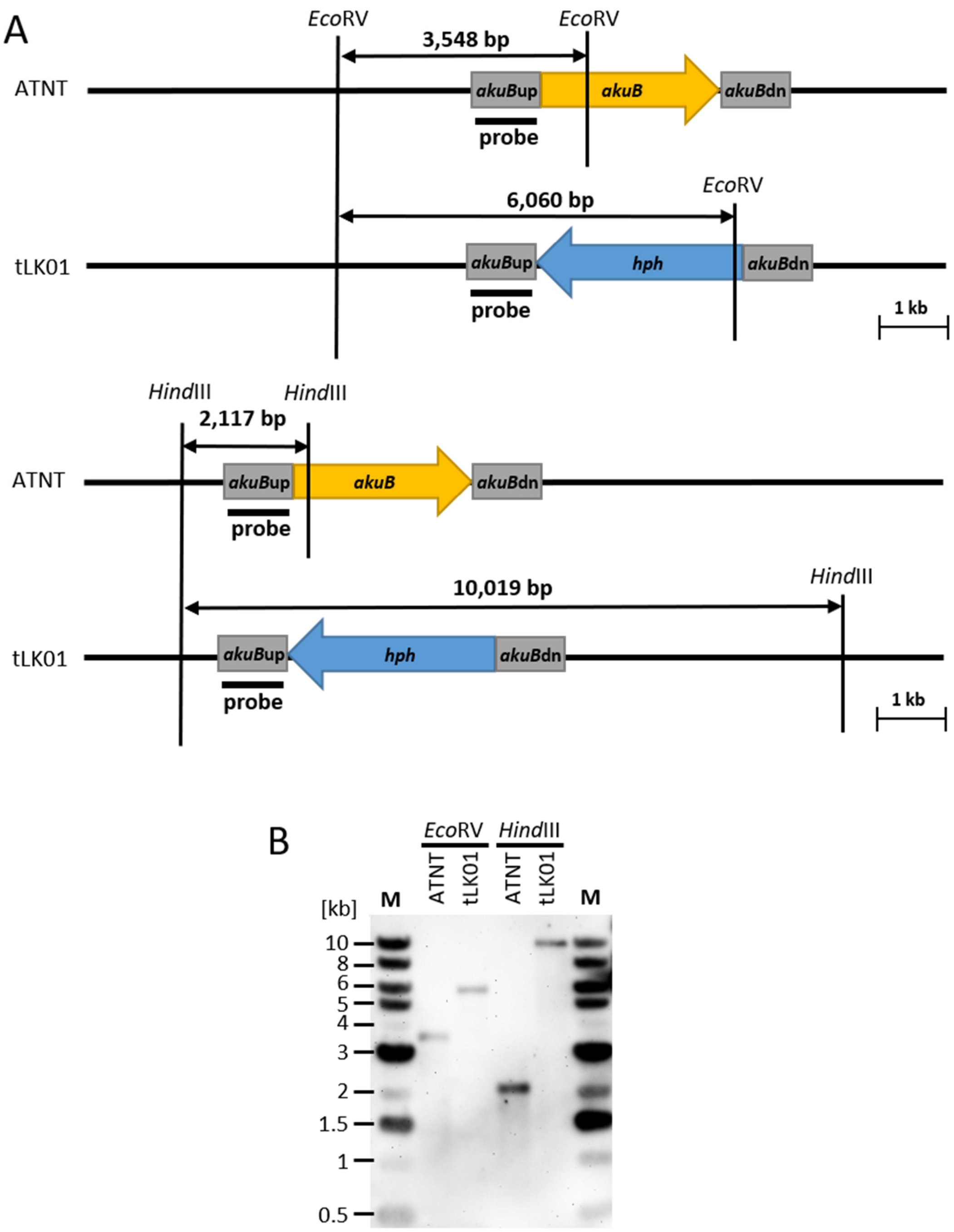

Figure S1. Southern Blot analysis for determination of the *akuB* deletion strain tLK01. A. Schematic representation of the genomic *akuB* locus of the strain ATNT and tLK01 (ATNTΔ*akuB*) with its respective *Eco*RV (upper panel) and *Hind*III restriction sites (lower panel). **B.** Southern Blot analysis of the *A. niger* parental strain ATNT and the *akuB* deletion strain tLK01**.** Genomic DNA was digested with *Eco*RV or *Hind*III. A digoxigenin-labeled probe was generated with oMG482/oMG483 to hybridize with *akuB* upstream sequence and signals were detected with CDPstar (Roche Diagnostics).
