# Supplementary material for: A genetic tool to express long fungal biosynthetic genes
Source: Fungal Biol Biotechnol. 2023 Feb 1;10:4. doi: 10.1186/s40694-023-00152-3 (PMC9893682; doi:10.1186/s40694-023-00152-3)
Supplement: Supplementary file 6 — Additional file 6: Figure S2. Plasmid maps of the expression vectors. The plasmids pLK04 (A), pLK05 (B), pMG56 (C) and pMG58 (D) are based on the pSMX2-URA plasmid [1]. The gene fragments are: bla; β-lactamase (confers ampicillin resistance); calA1/5; 1 kb of the 5′or 3′ end of the calA gene; fwnAup and fwnAdown, 1 kb up- and downstream the A. niger fwnA polyketide synthase gene; URA-blaster (dark blue, contains several genes; see below); PterA, promoter of the terA terrein polyketide synthase gene of Aspergillus terreus; Tag, encodes the hexahistidin tag (and includes an SpeI and PacI site for insertion of the GOI); TtrpC, terminator of the trpC anthranilatsynthase component 2 gene of A. terreus; rep origin, origin of plasmid replication. The URA-blaster contains: PpyrG, promoter of the pyrG gene from Aspergillus nidulans; pyrG, orotidine 5′-phosphate decarboxylase gene from A. nidulans (confers uracil prototrophy); TpyrG, terminator of the pyrG gene from A. nidulans; and two prpB flanks that encode the methylcitrate synthase gene from Escherichia coli that facilitate a subsequent removal of the URA blaster cassette from the A. niger genome via a homologous recombination event (counter selection) by addition of 5-fluoroorotic acid [2]. [file 40694_2023_152_MOESM6_ESM.pdf]

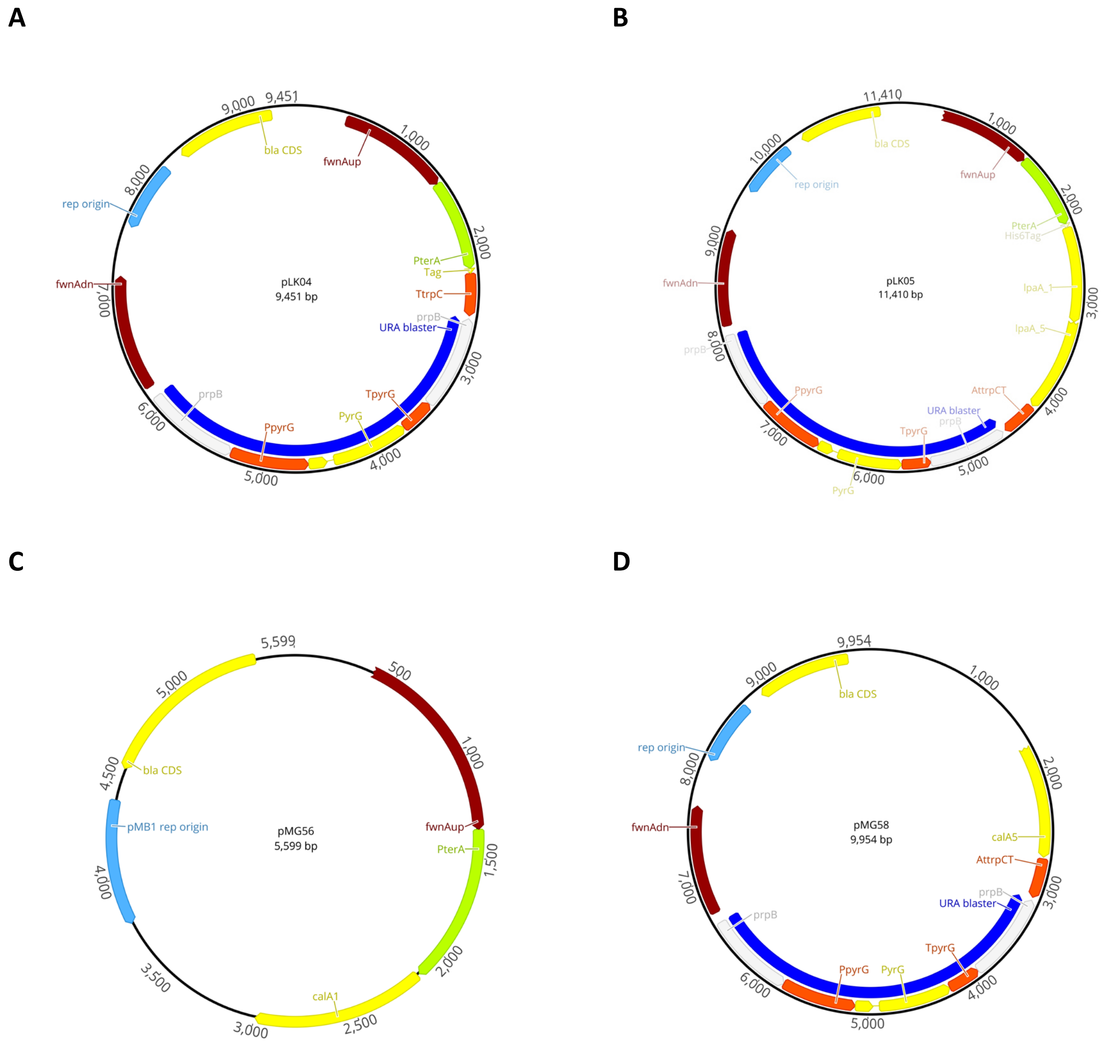

Figure S2. Plasmid maps of the expression vectors. The plasmids pLK04 (**A**), pLK05 (**B**), pMG56 (**C**) and pMG58 (**D**) are based on the pSMX2-URA plasmid [1]. The gene fragments are: *bla*; β-lactamase (confers ampicillin resistance); *calA1/5*; 1 kb of the 5´or 3´end of the *calA* gene; *fwnA*up and *fwnA*down, 1 kb up- and downstream the *A. niger fwnA* polyketide synthase gene; URA-blaster (dark blue, contains several genes; see below); P*terA*, promoter of the *terA* terrein polyketide synthase gene of *Aspergillus terreus*; Tag, encodes the hexahistidin tag (and includes an *SpeI* and *PacI* site for insertion of the GOI); T*trpC*, terminator of the *trpC* anthranilatsynthase component 2 gene of *A. terreus*; rep origin, origin of plasmid replication. The URA-blaster contains: P*pyrG*, promoter of the *pyrG* gene from *Aspergillus nidulans*; *pyrG*, orotidine 5´-phosphate decarboxylase gene from *A. nidulans* (confers uracil prototrophy); T*pyrG*, terminator of the *pyrG* gene from *A. nidulans*; and two *prpB* flanks that encode the methylcitrate synthase gene from *Escherichia coli* that facilitate a subsequent removal of the URA blaster cassette from the *A. niger* genome via a homologous recombination event (counter selection) by addition of 5-fluoroorotic acid [2].

Reference

1. Geib E, Brock M: **ATNT: an enhanced system for expression of polycistronic secondary metabolite gene clusters in *Aspergillus niger***. *Fungal Biol Biotechnol* 2017, **4**(1):e13.
2. Staab JF, Sundstrom P: **URA3 as a selectable marker for disruption and virulence assessment of *Candida albicans* genes**, *Trends in Microbiology*, 2003, **11**(2): 69-73.
