# Supplementary material for: A genetic tool to express long fungal biosynthetic genes
Source: Fungal Biol Biotechnol. 2023 Feb 1;10:4. doi: 10.1186/s40694-023-00152-3 (PMC9893682; doi:10.1186/s40694-023-00152-3)
Supplement: Supplementary file 7 — Additional file 7: Figure S3. Photographs of A. niger ATNT and tLK01 transformed with the fwnA deletion construct. Both pigmented and non-pigmented transformants have been detected in both experiments, but frequency of the homologous recombination into the fwnA locus is significantly higher in tLK01. For calculation of frequency of recombination see Additional file 2: Table S1. [file 40694_2023_152_MOESM7_ESM.pdf]

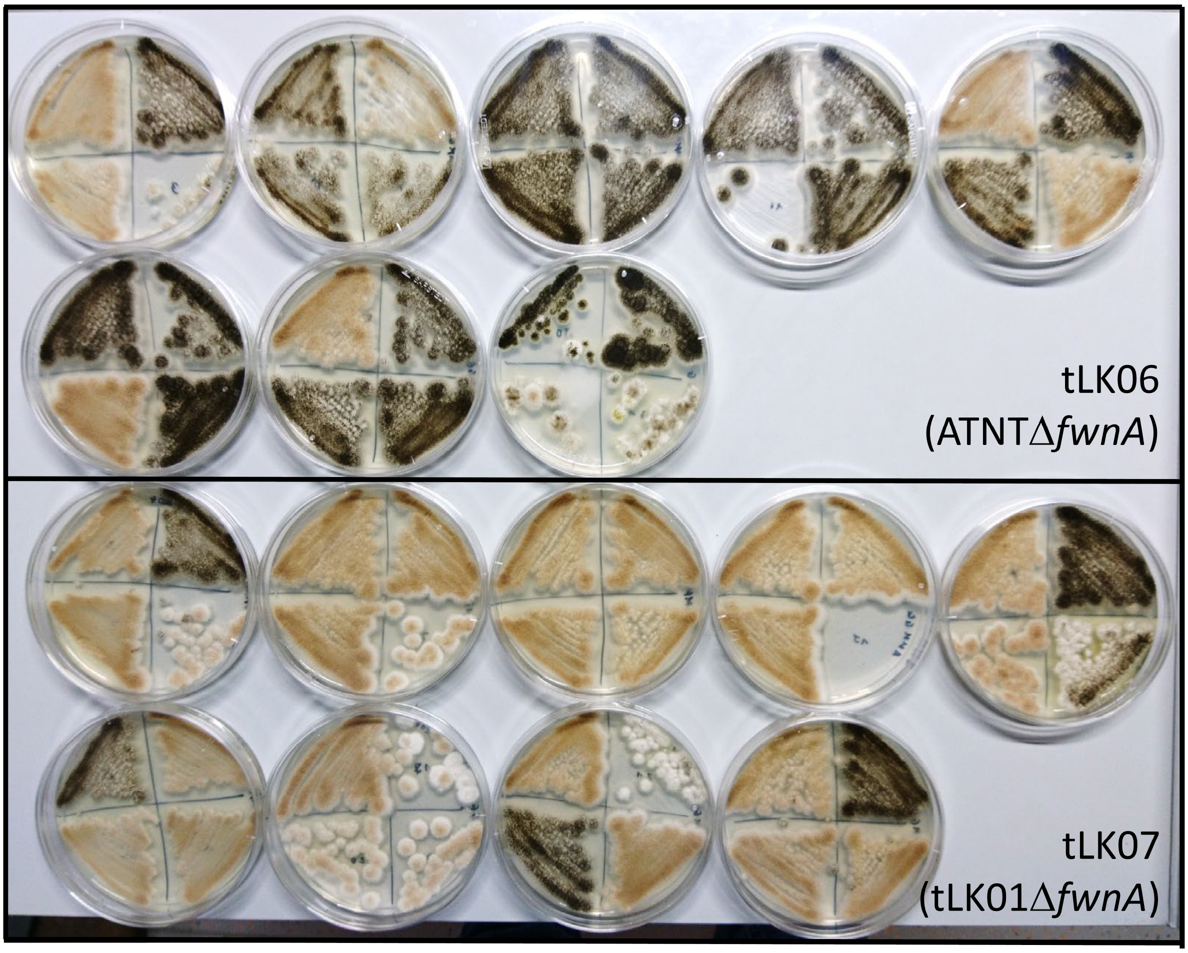

Figure S3. Photographs of *A. niger* ATNT and tLK01 transformed with the *fwnA* deletion construct. Both pigmented and non-pigmented transformants have been detected in both experiments, but frequency of the homologous recombination into the *fwnA* locus is significantly higher in tLK01. For calculation of frequency of recombination see Table S1.
