# Supplementary material for: A genetic tool to express long fungal biosynthetic genes
Source: Fungal Biol Biotechnol. 2023 Feb 1;10:4. doi: 10.1186/s40694-023-00152-3 (PMC9893682; doi:10.1186/s40694-023-00152-3)
Supplement: Supplementary file 8 — Additional file 8: Figure S4. Determination of the homologous integration of the PterA:TtrpC construct into the fwnA locus of recipient strains ATNT and tLK01. A. Schematic representation of the genomic fwnA locus of the parental strain ATNT and tLK01 (ATNT∆akuB) (upper lane) and the deletion mutants ATNT∆fwnA (tLK06) and tLK07 (ATNT∆akuB∆fwnA). B. Agarose gel of two diagnostic PCRs targeting the fwnA gene (upper lane) and the PterA promoter (lower lane). In contrast to the parental strains ATNT and tLK01, the non-pigmented ∆fwnA mutants (tLK06 and tLK07) lack the signal of the fwnA gene (upper panel). In lieu thereof, the homologous integration of PterA:TtrpC could be determined in the mutants (lower panel). [file 40694_2023_152_MOESM8_ESM.pdf]

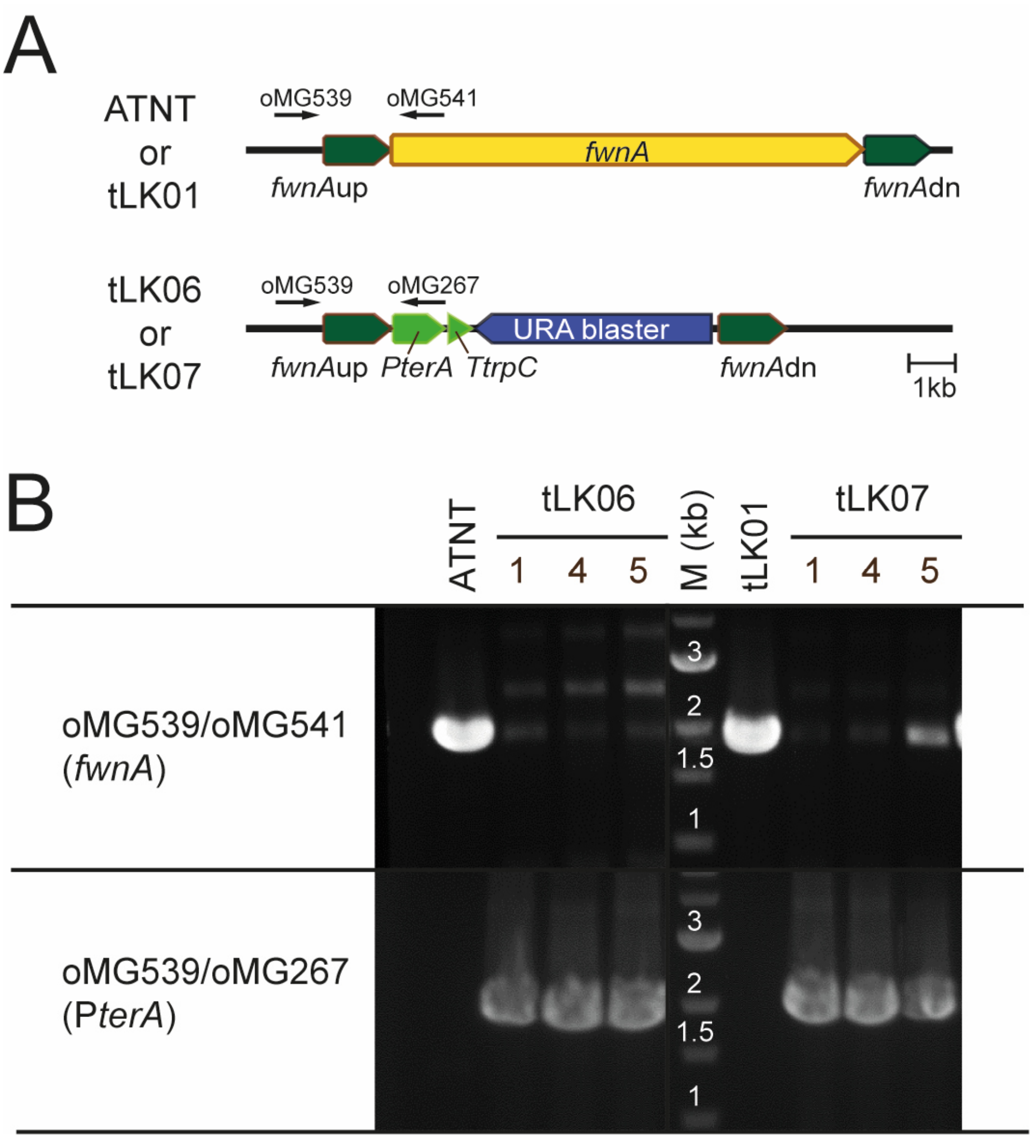

Figure S4. Determination of the homologous integration of the P*terA*:T*trpC* construct into the *fwnA* locus of recipient strains ATNT and tLK01. A. Schematic representation of the genomic *fwnA* locus of the parental strain ATNT and tLK01 (ATNTΔ*akuB*) (upper lane) and the deletion mutants ATNTΔ*fwnA* (tLK06) and tLK07 (ATNTΔ*akuB*Δ*fwnA*). **B.** Agarose gel of two diagnostic PCRs targeting the *fwnA* gene (upper lane) and the P*terA* promoter (lower lane). In contrast to the parental strains ATNT and tLK01, the non-pigmented Δ*fwnA* mutants (tLK06 and tLK07) lack the signal of the *fwnA* gene (upper panel). In *lieu* thereof, the homologous integration of P*terA*:T*trpC* could be determined in the mutants (lower panel).
