# Supplementary material for: A genetic tool to express long fungal biosynthetic genes
Source: Fungal Biol Biotechnol. 2023 Feb 1;10:4. doi: 10.1186/s40694-023-00152-3 (PMC9893682; doi:10.1186/s40694-023-00152-3)
Supplement: Supplementary file 9 — Additional file 9: Figure S5. Southern Blot analysis for determination of the fwnA deletion and lpaA overexpression in A. niger strains tLK04. A. Schematic representation of the genomic fwnA locus in the strain tLK01 (ATNT∆akuB) and the ∆fwnA::lpaA overexpression strain tLK04 with its respective SacII restriction sites. B. Southern Blot analysis of the A. niger parental strain tLK01 and five ∆fwnA::lpaA overexpression strains tLK04. Genomic DNA was digested with SacII. A digoxigenin-labeled probe was generated with oMG504/oMG505 to hybridize with the fwnA downstream sequence and signals were detected with CDPstar (Roche Diagnostics). Strains used for subsequent metabolic analysis are highlighted in green. [file 40694_2023_152_MOESM9_ESM.pdf]

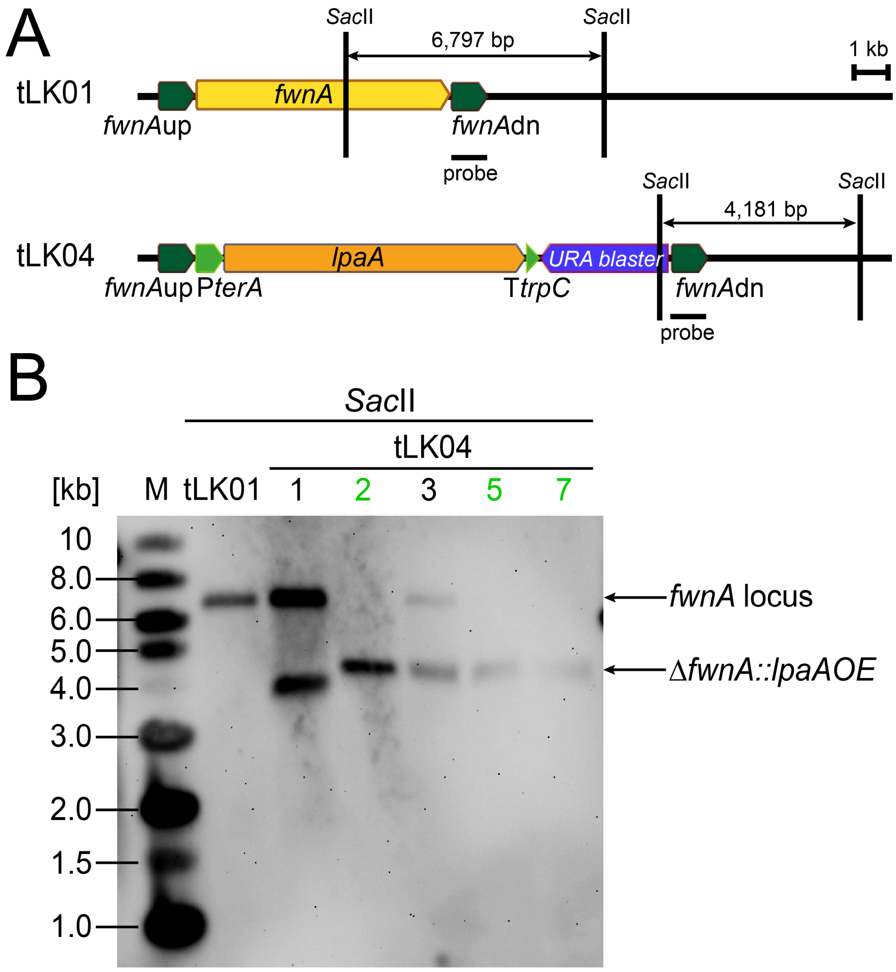

Figure S5. Southern Blot analysis for determination of the *fwnA* deletion and *lpaA* overexpression in *A. niger* strains tLK04. A. Schematic representation of the genomic *fwnA* locus in the strain tLK01 (ATNTΔ*akuB*) and the Δ*fwnA*::*lpaA* overexpression strain tLK04 with its respective *Sac*II restriction sites. **B.** Southern Blot analysis of the *A. niger* parental strain tLK01 and five Δ*fwnA*::*lpaA* overexpression strains tLK04**.** Genomic DNA was digested with *Sac*II. A digoxigenin-labeled probe was generated with oMG504/oMG505 to hybridize with the *fwnA* downstream sequence and signals were detected with CDPstar (Roche Diagnostics). Strains used for subsequent metabolic analysis are highlighted in green.
