# Supplementary material for: A genetic tool to express long fungal biosynthetic genes
Source: Fungal Biol Biotechnol. 2023 Feb 1;10:4. doi: 10.1186/s40694-023-00152-3 (PMC9893682; doi:10.1186/s40694-023-00152-3)
Supplement: Supplementary file 10 — Additional file 10: Figure S6. HR-MS and MS/MS spectra of laetiporic acids A1-D2 detectable in A. niger tLK04. High resolution MS/MS fragmentation of laetiporic acids A1/A2 (A), B1/B2 (B), C1/C2 (C) and D1/D2 (D) produced by A. niger tLK04. Indicated fragments are identical to the literature [1]. [file 40694_2023_152_MOESM10_ESM.pdf]

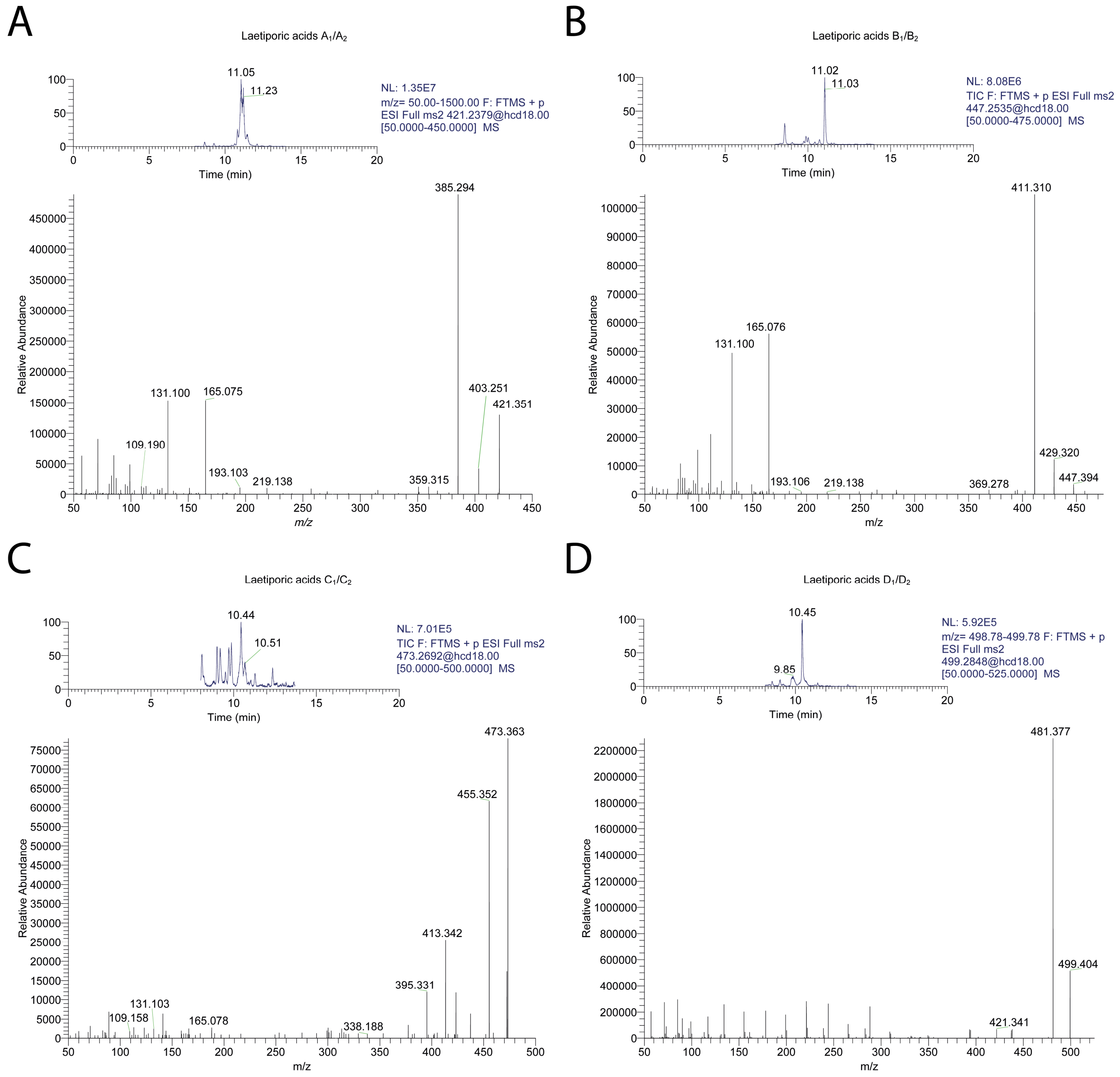

Figure S6. HR-MS and MS/MS spectra of laetiporic acids A_1-D_2 detectable in *A. niger* tLK04. High resolution MS/MS fragmentation of laetiporic acids A_1/A_2 (**A**), B_1/B_2 (**B**), C_1/C_2 (**C**) and D_1/D_2 (**D**) produced by *A. niger* tLK04. Indicated fragments are identical to the literature [1].

Reference

1. Seibold PS, Lenz C, Gressler M, Hoffmeister D: **The *Laetiporus* polyketide synthase LpaA produces a series of antifungal polyenes**. *J Antibiot (Tokyo)* 2020, **73**(10):711-720.
