# Supplementary material for: A genetic tool to express long fungal biosynthetic genes
Source: Fungal Biol Biotechnol. 2023 Feb 1;10:4. doi: 10.1186/s40694-023-00152-3 (PMC9893682; doi:10.1186/s40694-023-00152-3)
Supplement: Supplementary file 11 — Additional file 11: Figure S7. Expression of lpaA in the transgenic A. niger tLK07 (null mutant), tLK04 (lpaA expressing) and tLK05 (lpaAD1415A expressing). Expression was profiled by semi-quantitative PCR on the laetiporic acid synthase gene (lpaA) and referenced to the expression of the housekeeping gene encoding the glyceraldehyde-3-phosphate dehydrogenase (gpdA). RNA was isolated and cDNA was synthesized after cultivation for 36 h in inducing AMM (with doxycycline) at 30°C and 180 rpm. The genomic DNA (gDNA) of tLK07 or the lpaA-encoding plasmid pPS03 served as positive controls for gpdA and lpaA amplification, respectively. [file 40694_2023_152_MOESM11_ESM.pdf]

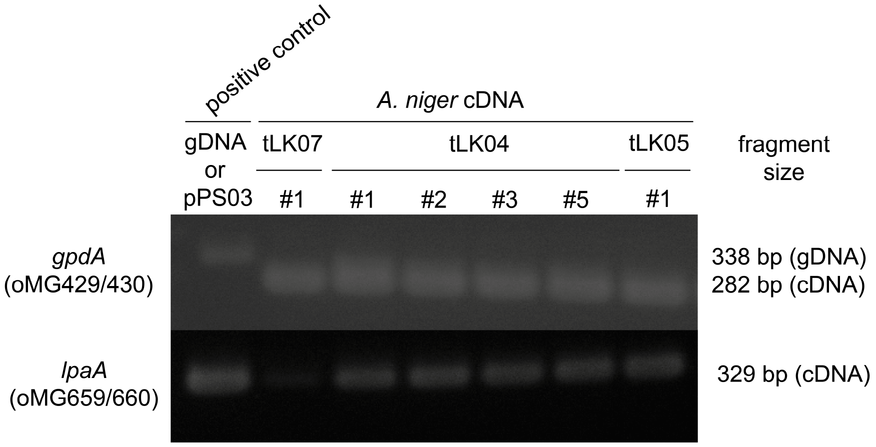

Figure S7. Expression of *lpaA* in the transgenic *A. niger* tLK07 (null mutant), tLK04 (*lpaA* expressing) and tLK05 (*lpaA*^{D1415A} expressing). Expression was profiled by semi-quantitative PCR on the laetiporic acid synthase gene (*lpaA*) and referenced to the expression of the housekeeping gene encoding the glyceraldehyde-3-phosphate dehydrogenase (*gpdA*). RNA was isolated and cDNA was synthesized after cultivation for 36 h in inducing AMM (with doxycycline) at 30°C and 180 rpm. The genomic DNA (gDNA) of tLK07 or the *lpaA*-encoding plasmid pPS03 served as positive controls for *gpdA* and *lpaA* amplification, respectively.
