# Supplementary material for: A genetic tool to express long fungal biosynthetic genes
Source: Fungal Biol Biotechnol. 2023 Feb 1;10:4. doi: 10.1186/s40694-023-00152-3 (PMC9893682; doi:10.1186/s40694-023-00152-3)
Supplement: Supplementary file 12 — Additional file 12: Figure S8. Integration of calA into the fwnA locus in A.niger tJMW06. A. Genomic locus of fwnA during recombination of the five calA DNA fragments in A. niger pJMW06. B. PCR amplification of four adjacent DNA fragment pairs was carried out using genomic DNA as templates. The A. niger parental strain tLK01 and the null mutant strain tLK07 (empty vector) served as negative controls. Three individual calA-expressing transformants tJMW06 #3, #13 and #26 showed the expected amplicon sizes of the recombined DNA fragments. [file 40694_2023_152_MOESM12_ESM.pdf]

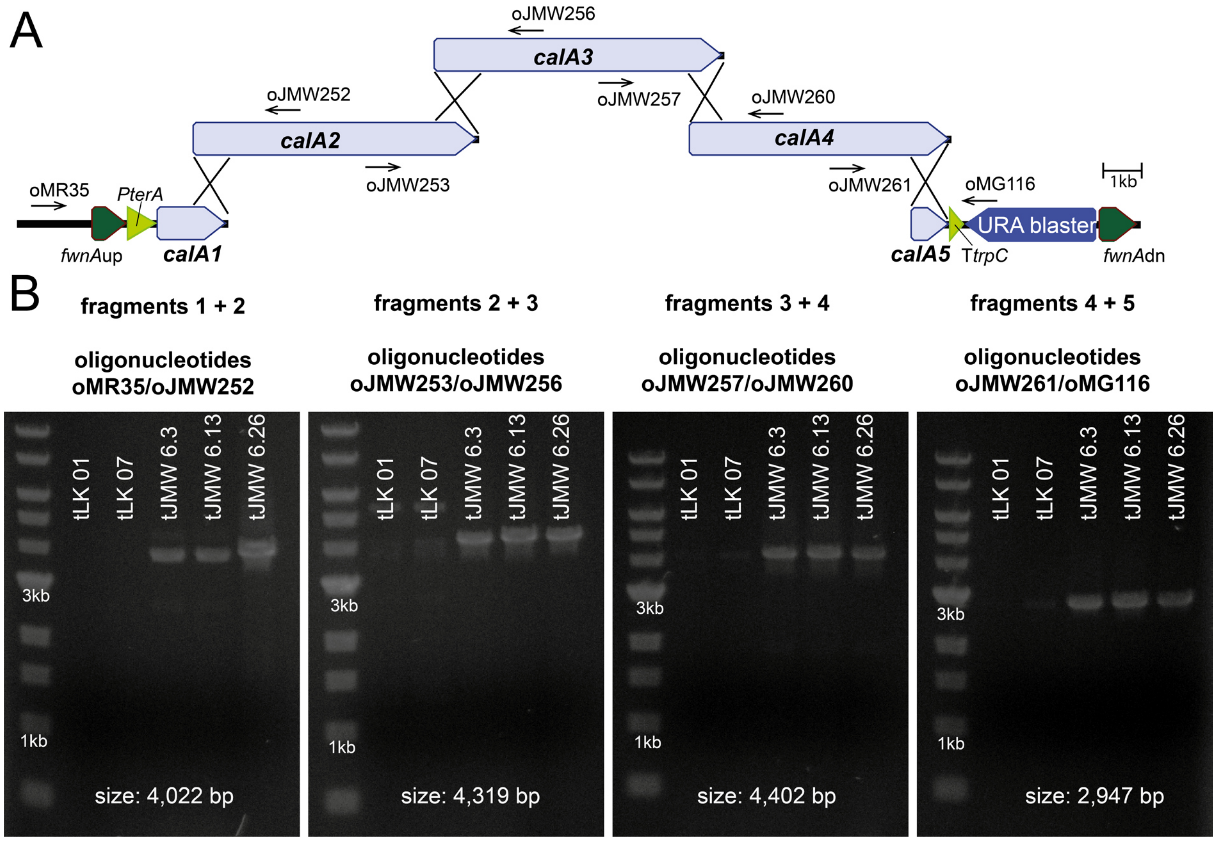

Figure S8. Integration of *calA* into the *fwnA* locus in *A.niger* tJMW06. **A.** Genomic locus of *fwnA* during recombination of the five *calA* DNA fragments in *A. niger* pJMW06. **B.** PCR amplification of four adjacent DNA fragment pairs was carried out using genomic DNA as templates. The *A. niger* parental strain tLK01 and the null mutant strain tLK07 (empty vector) served as negative controls. Three individual *calA*-expressing transformants tJMW06 #3, #13 and #26 showed the expected amplicon sizes of the recombined DNA fragments.
