# Supplementary material for: A genetic tool to express long fungal biosynthetic genes
Source: Fungal Biol Biotechnol. 2023 Feb 1;10:4. doi: 10.1186/s40694-023-00152-3 (PMC9893682; doi:10.1186/s40694-023-00152-3)
Supplement: Supplementary file 13 — Additional file 13: Figure S9. Southern Blot analysis for determination of the full-length calA integration into the genome of A. niger strain tJMW06. A. Schematic representation of the genomic fwnA locus in the strain tLK01 (ATNT∆akuB), the∆fwnA::calA overexpression locus of strain tJMW06.3 and the native calA locus of the calA gene donor strain M. alpina ATCC32222. B. Southern Blot analysis of the A. niger parental strain tLK01, the ∆fwnA::calA overexpression strain tJMW06 and M. alpina ATCC32222. Genomic DNA was double-digested with SmaI/DraI. A digoxigenin-labeled probe was generated with oMG569/oMG548 to hybridize with the fwnA downstream sequence and signals were detected with CDPstar (Roche Diagnostics). Full-length calA integration was determined for A. niger tJMW06 and directly reflects the size of the calA gene fragment in the gene donor strain M. alpina ATCC32222. [file 40694_2023_152_MOESM13_ESM.pdf]

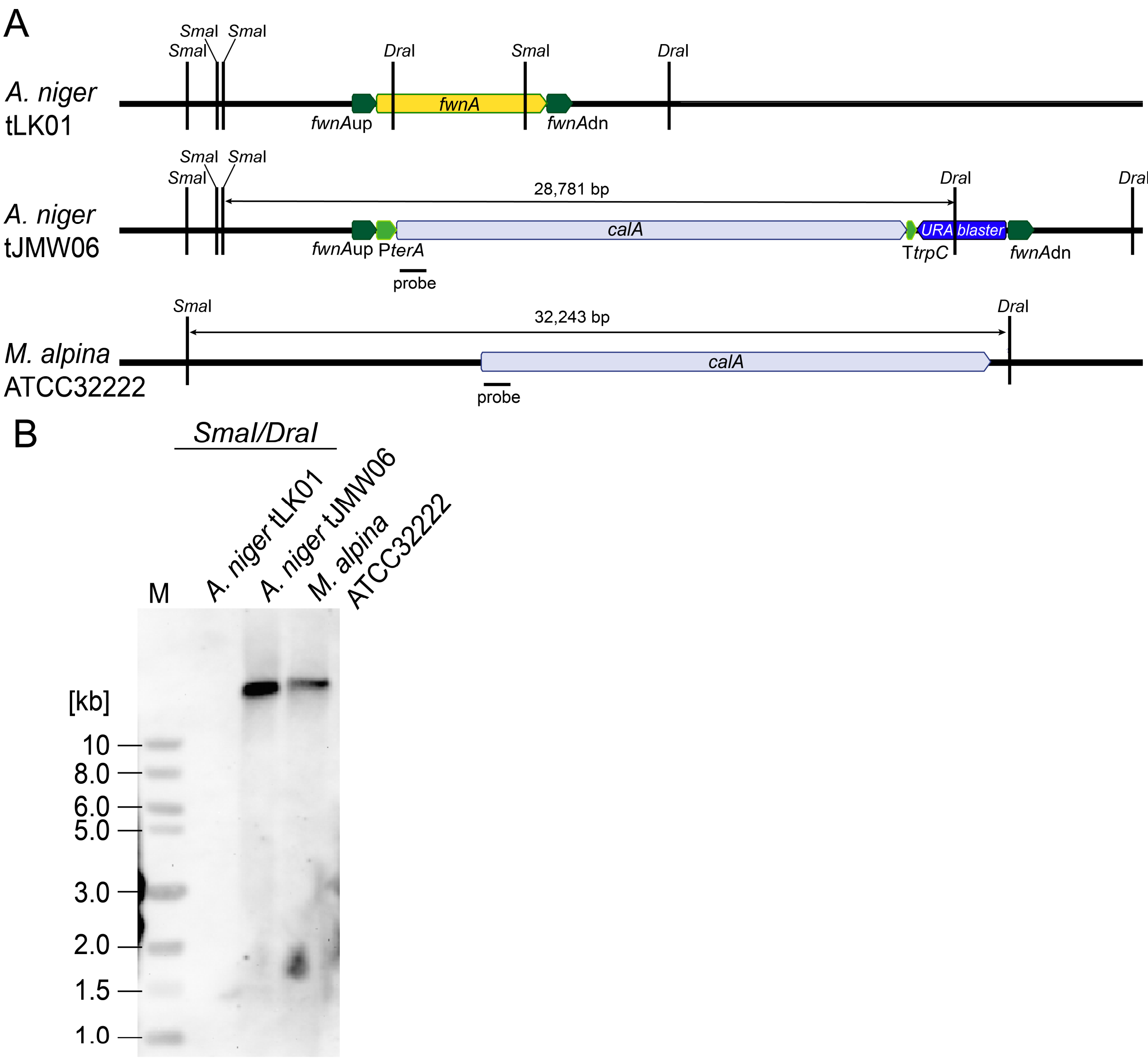


**Figure S9. Southern Blot analysis for determination of the full-length *calA* integration into the genome of *A. niger* strain tJMW06. A.** Schematic representation of the genomic *fwnA* locus in the strain tLK01 (ATNTΔ*akuB*), the Δ*fwnA*::*calA* overexpression locus of strain tJMW06.3 and the native *calA* locus of the *calA* gene donor strain *M. alpina* ATCC32222. **B.** Southern Blot analysis of the *A. niger* parental strain tLK01, the Δ*fwnA*::*calA* overexpression strain tJMW06 and *M. alpina* ATCC32222**.** Genomic DNA was double-digested with *Sma*I/*Dra*I. A digoxigenin-labeled probe was generated with oMG569/oMG548 to hybridize with the *fwnA* downstream sequence and signals were detected with CDPstar (Roche Diagnostics). Full-length *calA* integration was determined for *A. niger* tJMW06 and directly reflects the size of the *calA* gene fragment in the gene donor strain *M. alpina* ATCC32222.
