# Supplementary material for: A genetic tool to express long fungal biosynthetic genes
Source: Fungal Biol Biotechnol. 2023 Feb 1;10:4. doi: 10.1186/s40694-023-00152-3 (PMC9893682; doi:10.1186/s40694-023-00152-3)
Supplement: Supplementary file 14 — Additional file 14: Figure S10. Temperature dependent production of calpinactam in A. niger tJMW06 and M. alpina ATCC32222. Calpinactam production (bars) and total fungal dry weight (boxes) are indicated for several cultivation conditions. The transformant A. niger tJMW06 was cultivated in YPD with 30 µg mL1 doxycycline as inducer at 20, 25 and 30°C for 3 days. The calA gene donor strain M. alpina ATCC32222 was cultivated in MEP (25°C) for 4 days. Production rate in A. niger tJMW06 is optimal at 25 °C, which is the growth optimum for M. alpina. Experiments were carried in triplicate. [file 40694_2023_152_MOESM14_ESM.pdf]

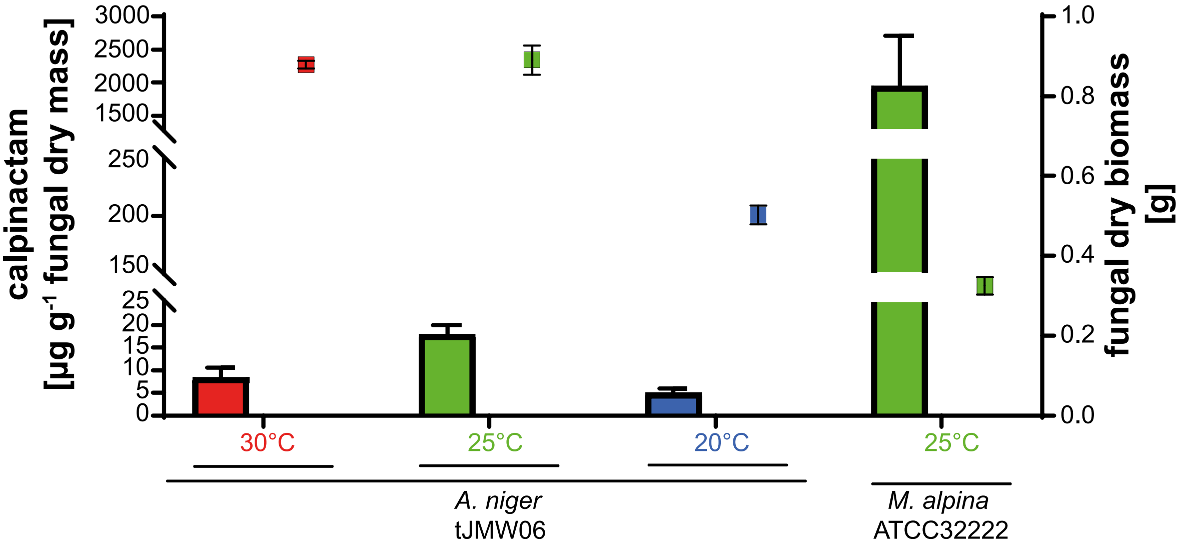

Figure S10. Temperature dependent production of calpinactam in *A. niger* tJMW06 and *M. alpina* ATCC32222*.* Calpinactam production (bars) and total fungal dry weight (boxes) are indicated for several cultivation conditions. The transformant *A. niger* tJMW06 was cultivated in YPD with 30 µg mL^{-1} doxycycline as inducer at 20, 25 and 30°C for 3 days. The *calA* gene donor strain *M. alpina* ATCC32222 was cultivated in MEP (25°C) for 4 days. Production rate in *A. niger* tJMW06 is optimal at 25°C, which is the growth optimum for *M. alpina.* Experiments were carried in triplicate.
